# Large‐scale distribution of microbial and viral populations in the South Atlantic Ocean

**DOI:** 10.1111/1758-2229.12381

**Published:** 2016-02-16

**Authors:** Daniele De Corte, Eva Sintes, Taichi Yokokawa, Itziar Lekunberri, Gerhard J. Herndl

**Affiliations:** ^1^ University of ViennaDepartment of Limnology and Bio‐OceanographyCenter of EcologyAlthanstrasse 141090ViennaAustria; ^2^ Center for Marine Environmental Studies (CMES)Ehime UniversityBunkyo 3Ehime790‐8577Japan; ^3^ Department of Biological OceanographyRoyal Netherlands Institute for Sea Research (NIOZ)PO Box 591790 ABDen BurgThe Netherlands; ^4^ Present address: Marine Functional Biology GroupResearch and Development Center for Marine BiosciencesJapan Agency for Marine‐Earth Science and Technology (JAMSTEC)Natushima 2‐15YokosukaKanagawa237‐0061Japan

## Abstract

Viruses are abundant, diverse and dynamic components of the marine environments and play a significant role in the ocean biogeochemical cycles. To assess potential variations in the relation between viruses and microbes in different geographic regions and depths, viral and microbial abundance and production were determined throughout the water column along a latitudinal transect in the South Atlantic Ocean. Path analysis was used to examine the relationships between several abiotic and biotic parameters and the different microbial and viral populations distinguished by flow cytometry.

The depth‐integrated contribution of microbial and viral abundance to the total microbial and viral biomass differed significantly among the different provinces. Additionally, the virus‐to‐microbe ratio increased with depth and decreased laterally towards the more productive regions. Our data revealed that the abundance of phytoplankton and microbes is the main controlling factor of the viral populations in the euphotic and mesopelagic layers, whereas in the bathypelagic realm, viral abundance was only weakly related to the biotic and abiotic variables. The relative contribution of the three viral populations distinguished by flow cytometry showed a clear geographical pattern throughout the water column, suggesting that these populations are composed of distinct taxa able to infect specific hosts. Overall, our data indicate the presence of distinct microbial patterns along the latitudinal transect. This variability is not limited to the euphotic layer but also detectable in the meso‐ and bathypelagic layers.

## Introduction

Microbes are the most abundant and genetically diverse life forms on Earth (Pace, 1997; Rappe and Giovannoni, 2003; Riesenfeld *et al*., 2004) and the oceans cover more than 70% of the Earth's surface. Thus, marine microorganisms account for more than 90% of the total ocean's biomass and are the main mediators of the marine biogeochemical cycles (Azam *et al*., 1983; Di Poi *et al*., 2013). In surface waters, viruses are about 10‐fold more abundant than prokaryotes (Parada *et al*., 2007; 2008). However, they account for only 5% of the total marine biomass because of their small size (Suttle, 2005). Together with flagellates, viruses are the major cause of microbial and phytoplankton mortality (Weinbauer *et al*., 2003; Brussaard, 2004a; Pernthaler, 2005). Hence, viruses play a major role in controlling key components of the microbial food web and in stimulating dissolved organic matter cycling via cell lysis and the associated release of intracellular material from the host cells (Middelboe *et al*., 1996; Middelboe and Lyck, 2002).

Viruses lack universally conserved marker genes (such as the 16S rRNA gene for prokaryotes). Thus, viral diversity only started to be explored upon the advancement of –omics approaches (Edwards and Rohwer, 2005; Angly *et al*., 2006). Generally, viral abundance co‐varies with the host abundance and activity. Several studies have shown, however, that virus‐to‐microbe ratios (VMRs) vary with the geographical location and depth in the oceanic water column (Parada *et al*., 2007; De Corte *et al*., 2012; Yang *et al*., 2014). This suggests that in marine systems, the viral–microbial interactions and their life strategies (lytic versus lysogenic) may change with changing environmental conditions. Surprisingly, the decrease in viral abundance towards the more oligotrophic regions and deeper layers of the ocean is less pronounced than the decrease in microbial abundance leading to higher VMRs in these areas (Parada *et al*., 2007; De Corte *et al*., 2012; Yang *et al*., 2014). These varying VMRs are in contrast with the common notion that the abundance of viruses is tightly linked to microbial abundance and activity. Thus, in the dark and in oligotrophic areas of the ocean, the trade‐off between viral production and decay may be substantially distorted, leading to higher VMRs than in mesotrophic surface waters. Consequently, it remains enigmatic which survival and reproduction (lytic versus lysogenic) mechanisms of viruses prevail under different trophic conditions in the ocean. It has been proposed, however, that under oligotrophic conditions, the lysogenic life cycle of viruses prevails, whereas under more eutrophic conditions the lytic cycle dominates (Weinbauer and Suttle, 1999; Weinbauer *et al*., 2003).

The aim of this study was to examine the distribution of different viral populations distinguished by flow cytometry along a latitudinal transect in the South Atlantic Ocean and to identify the main controlling factors determining the microbial and viral distribution pattern among different depth layers. Path analysis was used to disentangle the effect of the different environmental variables on the virus–microbe interactions.

## Materials and methods

### Study area and sampling

**Figure 1:**
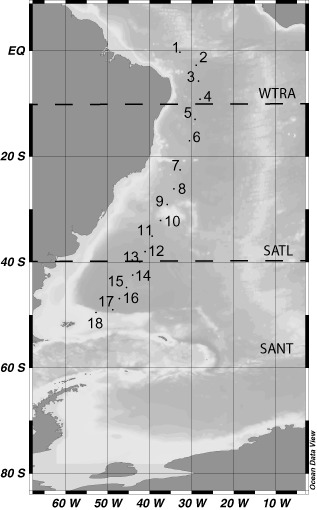
Location of the stations (indicated by dots) sampled in the South Atlantic Ocean during the GEOTRACES (leg‐3) cruise covering three oceanic provinces (Longhurst, 1998): Western Tropical Atlantic (WTRA) (0°S–10°S), the South Atlantic Gyral (SATL) (10°S–40°S) and the Subantarctic province (SANT) (40°S–55°S).

Water samples were collected at 24 depth layers at 18 stations during the GEOTRACES leg‐3 cruise (April 2011) in the South Atlantic Ocean on board R/V *James Cook* (Fig. 1). The study area was divided in three different oceanic provinces: the Western Tropical Atlantic (WTRA) (0°S–10°S), the South Atlantic Gyral (SATL) (10°S–40°S) and the Subantarctic province (SANT) (40°S–55°S) comprising the Subtropical Convergence Zone (40°S–45°S) and the Subantarctic Water Ring (45°S–55°S) (Longhurst, 1998).

Sampling was performed with a rosette sampler equipped with 25L Niskin bottles, a conductivity‐temperature‐depth system (SBE43 Seabird, Bellevue, WA, USA) and a dissolved oxygen sensor.

### Microbial and viral abundance

The microbes distinguished by flow cytometry with the current protocol consist mainly of bacterial and archaeal cells.

Samples for microbial and viral abundance were collected at all the 18 stations at 24 depth layers (Table S1). Flow cytometry after nucleic acid staining was used to enumerate both viruses and microbes as described elsewhere (Del Giorgio *et al*., 1996; Marie *et al*., 1999; Brussaard, 2004b).

Depending on their respective signature in the cytogram of green fluorescence versus side scatter, two different microbial populations [high nucleic acid content microbes (HNA) and low nucleic acid content microbes (LNA)] and three different viral populations [high nucleic acid content viruses (V_HNA), medium nucleic acid content viruses (V_MNA) and low nucleic acid content viruses (V_LNA)] were discriminated.

The depth‐integrated microbial and viral abundances were calculated using the equation:

$$\sum_{i=1}^{i=n}\frac{\left(A_{i}+A_{i-1}\right)*\left(D_{i}-D_{i-1}\right)}{2}$$

where *A* is the microbial or viral abundance and *D* is the depth (*i* is the index of summation and *n* is the upper limit of summation). Their contribution in the epipelagic (5–200 m), mesopelagic (200–1000 m) and bathypelagic (1000 – about 100 m above bottom) to the integrated abundance over the entire water column was calculated as:

$$\left(\frac{integrated\;abundance\;at\;a\;specific\;depth\;layer}{integrated\;abundance\;of\;the\;entire\;water\;column}\right)\times 100$$

### Picophytoplankton abundance

Samples for picophytoplankton abundance were collected at all the stations in the epipelagic layer (5–250 m). Fluorescing photosynthetic pigments were used to enumerate the different picophytoplankton populations by flow cytometry. *Synechococcus*, *Prochlorococcus* and photosynthetic picoeukaryotes were distinguished according to their respective signature in the cytogram of red fluorescence versus side scatter and versus orange fluorescence (Olson *et al*., 1993).

### Leucine incorporation rates by heterotrophic microbes

Samples to measure leucine incorporation by heterotrophic microbes were collected at eight depths (Table S1) including the main three (epi‐, meso‐ and bathypelagic) pelagic zones at 15 stations. Microbial leucine incorporation was measured on triplicate 10–40 ml samples (depending on the expected activity) and on triplicate formaldehyde‐killed blanks (Simon and Azam, 1989). The samples and blanks were inoculated with 5 nM ^3^H‐leucine (final concentration, specific activity 160 Ci mmol^−1^) and incubated in the dark at in situ temperature for 4–24 h depending on the expected activity. Subsequently, the samples were fixed with formaldehyde (2% final concentration), filtered onto 0.2 μm polycarbonate GTTP filters (Millipore, Billerica, MA, USA) supported by Millipore HAWP filters and rinsed three times with 10 ml of 5% ice‐cold trichloroacetic acid. Thereafter, the filters were transferred into scintillation vials and dried at room temperature. Then, 8 ml of scintillation cocktail (Packard Filter Count, Perkin‐Elmer, Waltham, MA, USA) was added to each vial and the vials were counted in a Tri‐Carb 2910TR (Perkin‐Elmer, Groningen, The Netherlands) liquid scintillation counter after 18 h. The obtained disintegrations per minute were converted to leucine incorporation rates. The cell‐specific leucine incorporation rate was calculated by dividing the bulk leucine incorporation rates by the respective microbial abundance (Kirchmam, 2001).

### Particulate organic carbon (POC) flux

The POC fluxes were estimated using the equation given elsewhere (Antia *et al*., 2001) relating primary production (g C m^−2^ per year) to the POC flux at a given depth (m), Z:

$$\text{Fz}\;\left({\text{g C m}}^{-2}{\text{yr}}^{-1}\right)=0.1\times {\left(\text{primary production}\right)}^{1.77}\times {\left(Z\right)}^{-0.68}$$

The primary production values used in this equation were retrieved from the Ocean Productivity website (http://www.science.oregonstate.edu/ocean.productivity/); handling of the data is described elsewhere (Yokokawa *et al*., 2013).

### Statistical analysis

Because our data were not normally distributed (Shapiro–Wilk normality test), non‐parametric statistical tests were used. Hence, Spearman rank correlation was performed to analyse the relationship between several measured parameters and analysis of variance (ANOVA on rank) was performed to test for possible differences among depth layers and geographic provinces. Regression analysis was used to predict the relationship between the log‐transformed microbial abundance and production versus log‐transformed POC flux. Path analysis was used to explain the linear relationships (R^2^ = coefficient of determination) between the biological and the contextual physicochemical parameters. Path analysis was conducted as a hierarchical multiple regression analysis, where R^2^ is the coefficient of determination of the linear regression and the beta weights are the regression coefficients for the specific standardized independent variables. The ratio of the beta weights is the ratio of the predictive importance of the independent variables. The obtained results were used to establish a path diagram (SPSS Amos). This analysis was also applied to the data obtained at GEOTRACES leg‐1 and ‐2 given in De Corte and colleagues (2012).

## Results

### Physical and chemical parameters

Water temperature in the individual depth layers decreased from the WTRA to the SANT province. This decrease in temperature was not limited to the surface waters, but was also detectable in the meso‐ and bathypelagic layers (Table S2). In the mesopelagic layers, the highest temperature was detected in the SATL province (Table S2). Salinity showed a similar trend as temperature, decreasing towards the higher latitudes from 35.23 ± 0.73 in the WTRA to 34.50 ± 0.30 in the SANT province (Table S2). Moreover, the highest mesopelagic salinity was recorded in the SATL province.

The apparent oxygen utilization (AOU) varied with depth and latitude. AOU amounted to 115.82 ± 25.18 μmol kg^−1^ in the bathypelagic waters (Table S2). The AOU of the mesopelagic layers was higher in the WTRA and in the SANT than in the SATL province (Table S2). The AOU in the bathypelagic layers significantly increased towards the high latitudes (ANOVA on rank, *P* < 0.01) (Table S2).

### Microbial and viral abundance

**Figure 2:**
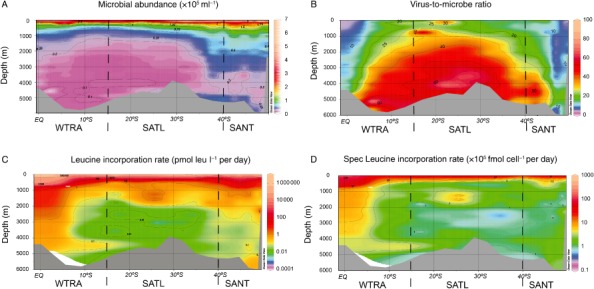
Microbial parameters measured in the South Atlantic Ocean throughout the water column: (A) microbial abundance (MA), (B) virus‐to‐microbe ratio (VMR), (C) microbial heterotrophic production (MHP) and (D) cell‐specific leucine incorporation rate.

The abundance of microbes decreased with depth, from an average of 3.6 ± 2.1 × 10^5^ cells ml^−1^ in the epipelagic layer to 0.24 ± 0.14 × 10^5^ cells ml^−1^ in the bathypelagic layer (ANOVA on rank, *P* < 0.01) (Table S3, Fig. 2A). Additionally, microbial abundance significantly increased towards the higher latitudes from the WTRA to the SANT province (ANOVA on rank, *P* < 0.01). This increase was not limited to the surface layers but was detectable throughout the entire water column (Table S3, Fig. 2A). Microbial abundance was related to the POC flux estimated from phytoplankton primary production (r^2^ = 0.63, *P* < 0.01) (Fig. S1).

**Figure 3:**
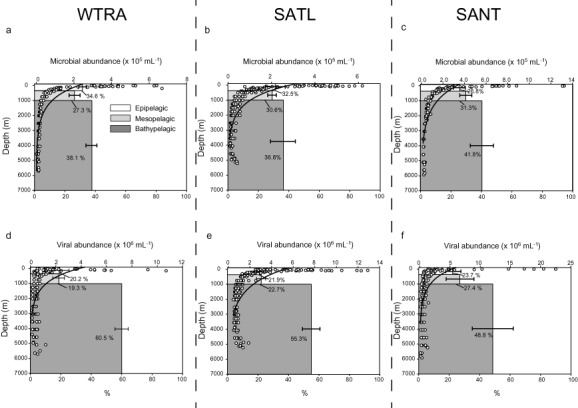
Average integrated microbial (A–C) and viral (D–F) abundance (average and % of total abundance) in the epi‐, meso‐ and bathypelagic layers of the different oceanic provinces WTRA (A, D), SATL (B, E) and SANT (C, F). The open dots represent the microbial and viral abundances with the best‐fit regression line through the data points.

Combining the data from the three oceanic provinces, we found the integrated microbial abundance of the epipelagic, mesopelagic and bathypelagic layers accounted for 31% ± 4%, 29% ± 2% and 39% ± 3% of the total microbial abundance respectively. Whereas the integrated microbial abundance of the epipelagic realm decreased from WTRA to SANT, i.e., towards high latitudes, in the mesopelagic layers it increased towards high latitudes (ANOVA on rank *P* < 0.01) (Fig. 3A, B, C).

The HNA, distinguished based on their signature in a plot of green fluorescence versus side scatter, increased in relative abundance with depth, from an average of 49.9% ± 6.9% in the epipelagic to 58.4% ± 5.4% in the deepest layers (r^2^ = 0.38, *P* < 0.001) (Fig. S2). The relative contribution of HNA to total microbial abundance did not show any significant trend with latitude (Table S3). The percentage of HNA was negatively correlated with temperature, microbial heterotrophic activity and viral abundance (Table 1).

**Table 1:** Spearman's rank correlation coefficients between different environmental and biological parameters

	Temp (°C)	Depth (m)	PA	% HNA	VA	% V_HNA	% V_MNA	% V_LNA	MPR	aH‐Leu	Spec aH‐Leu	POC	Prochlorococcus	Synechococcus	Picoeukaryotes
Temp (°C)	–														
Depth (m)	**−0.94**	–													
MA	**0.80**	**−0.91**	–												
% HNA	**−0.71**	**0.61**	**−0.49**	–											
VA	**0.68**	**−0.71**	**0.74**	**−0.45**	–										
% V_HNA	**0.80**	**−0.85**	**0.83**	**−0.51**	**0.21**	–									
% V_MNA	**0.20**	**−0.29**	**0.33**	−0.12	−0.08	**0.59**	–								
% V_LNA	**−0.31**	**0.39**	**−0.42**	**0.18**	0.01	**−0.78**	**−0.95**	–							
VMR	**−0.39**	**0.51**	–	**0.18**	‐	**0.45**	**−0.61**	**0.62**	–						
aH‐Leu	**0.71**	**−0.81**	**0.85**	**−0.43**	**0.55**	**0.53**	**0.50**	**−0.58**	**−0.66**	–					
Spec aH‐Leu	**0.50**	**−0.54**	‐	−0.22	**0.32**	**0.48**	**0.33**	**−0.45**	**−0.38**	–	–				
POC	**0.39**	**−0.62**	**0.80**	**−0.16**	**0.49**	**0.37**	**0.44**	**−0.45**	**−0.63**	**0.62**	**0.32**	–			
Prochlorococcus	**0.72**	**−0.59**	**0.45**	−0.07	**0.43**	**0.40**	**−0.24**	0.02	0.13	−0.21	−0.04	−0.13	–		
Synechococcus	**0.34**	**−0.75**	**0.79**	−0.07	**0.50**	**0.30**	0.13	**−0.25**	−0.16	−0.16	0.01	**0.47**	**0.59**	–	
Picoeukaryotes	**0.22**	**−0.52**	**0.65**	**−0.32**	**0.52**	**0.42**	−0.03	−0.15	0.37	0.32	0.13	**0.20**	**0.61**	**0.57**	–

Statistically significant correlation coefficients (*P*‐value < 0.05) are marked in bold.

a H‐Leu, leucine incorporation rate; HNA, high nucleic acid content microbes; MA, microbial abundance; SPEC_^3^H‐Leu, specific leucine incorporation rate; VA, viral abundance; VMR, viral to microbial ratio; V_HNA, high nucleic acid content viruses; V_LNA, low nucleic acid content viruses; V_MNA, medium nucleic acid content viruses.

**Figure 4:**
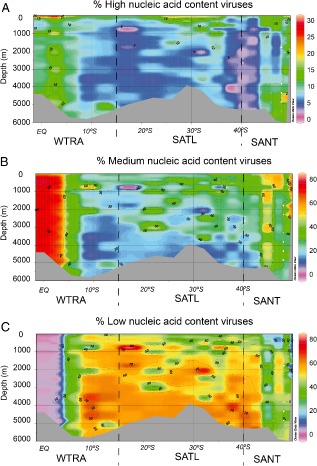
Relative contribution of the three viral populations to the total viral abundance throughout the water column along the South Atlantic latitudinal transect: (A) HNA, (B) MNA and (C) LNA viruses.

Viral abundance exponentially decreased with depth from 46.6 ± 40.6 × 10^5^ viruses ml^−1^ in epipelagic waters to 6.1 ± 3.1 × 10^5^ viruses ml^−1^ in the bathypelagic realm. Considering the whole water column, we found that viral abundance was positively related to microbial abundance and heterotrophic microbial production. Considering the epipelagic realm separately, we also found a positive correlation of viral abundance to total picophytoplankton abundance (Table 1, Fig. S3). All three viral populations (V_HNA, V_MNA and V_LNA) distinguished by flow cytometry decreased in abundance with depth (ANOVA on rank, *P* < 0.01) without a significant geographical trend (Fig. S4). However, the relative contribution of the three different populations to the total viral abundance showed a clear geographic pattern (Fig. 4). The percentages of V_HNA and V_MNA viruses were higher in the WTRA and in the SANT province throughout the entire water column than in the SATL. Conversely, the percentage of the V_LNA population was higher in the SATL province than in the WTRA and SANT (Fig. 4, Table S3).

The V_HNA and V_MNA populations were positively correlated with leucine incorporation rates and bacterial abundance, whereas the V_LNA population was negatively correlated with both leucine incorporation rates and microbial abundance. Also, the V_HNA population of the epipelagic layer was positively correlated with total picophytoplankton abundance and negatively correlated with depth (Table 1).

The VMR significantly increased with depth from an average of 15.0 ± 15.7 in the epipelagic layer to 32.9 ± 25.1 in the bathypelagic layer (ANOVA on rank, *P* < 0.01) (Fig. 2B, Table S3). Moreover, the VMR varied according to the geographic location, exhibiting the lowest values in the WTRA and SATL province (ANOVA on rank, *P* < 0.01) (Table S3, Fig. 2B). The VMR was negatively correlated with leucine incorporation rates, temperature and with the POC flux indicating the overall depth‐related increase in VMR (Table 1, Fig. 2B).

The integrated viral abundance over the three different provinces of the epipelagic, mesopelagic and the bathypelagic realms contributed 22% ± 2%, 23% ± 4% and 55% ± 6% to the total viral abundance respectively. The integrated viral abundance increased in the epi‐ and mesopelagic layers towards the SANT province, whereas in the bathypelagic layers it decreased (ANOVA on rank *P* < 0.01) in contrast to the microbial abundance (Fig. 3D–F).

### Leucine incorporation rates

Leucine incorporation, as a proxy of heterotrophic microbial production, decreased with depth from an average of 278 ± 141 pmol leu l^−1^ per day to 0.62 ± 0.91 pmol leu l^−1^ per day. Significant differences in leucine incorporation rates were also found between the different geographic regions throughout the water column. The geographic variation was not limited to the surface layer but was also found in the meso‐ and bathypelagic layers (Table S3, Fig. 2C). Overall, the leucine incorporation rate was also significantly related to the POC flux [from the meso‐ to the bathypelagic layers (r^2^ = 0.34, *P* < 0.01)] (Fig. S1).

The specific leucine incorporation rate decreased from the epipelagic layer (61 ± 33 × 10^−5^ fmol cell^−1^ per day) to the bathypelagic layer (2.56 ± 4.28 × 10^−5^ fmol cell^−1^ per day) (ANOVA on rank *P* < 0.01). The specific leucine incorporation rate showed a similar geographic pattern as bulk leucine incorporation (Fig. 2C, D).

### Links between biological and environmental variables

**Figure 5:**
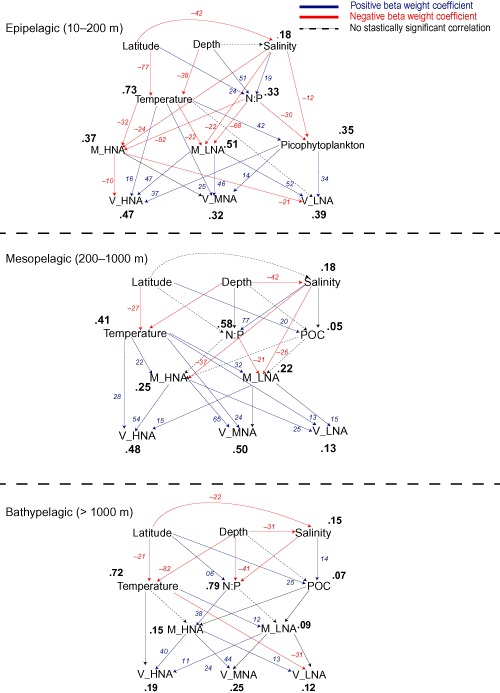
Path diagrams showing the hypothesized relationships between the biotic and abiotic parameters and the microbial, viral and picophytoplankton compartments in the Atlantic Ocean in the three depth layers (epi‐, meso‐ and bathypelagic). In italics (red or blue) the path coefficients (beta weights) leading to the coefficients of determination (R^2^) in bold.

In the epipelagic layer, the variability in picophytoplankton abundance was largely explained by temperature, nutrient concentrations and salinity (r^2^ = 0.35, *P* < 0.01) as well as by the percentage of HNA and LNA (r^2^ = 0.37, r^2^ = 0.51, *P* < 0.01, respectively) (Fig. 5). The variability of the three viral populations (high, medium and low fluorescence) in the epipelagic layer was largely explained by microbial abundance and temperature (r^2^ = 0.47, r^2^ = 0.32, r^2^ = 0.39, *P* < 0.01, respectively). Conversely, in the mesopelagic layer, the variation in the percentage of the high and low nucleic acid containing microbial populations was only partially explained by the environmental parameters (r^2^ = 0.25, r^2^ = 0.22, respectively, *P* < 0.01). The variation in the V_HNA and V_MNA viral populations was largely explained by host abundance and temperature (r^2^ = 0.48, r^2^ = 0.50, *P* < 0.01, respectively) in the mesopelagic environment (Fig. 5), but not the variation of the LNA viral population (r^2^ = 0.13). In the bathypelagic layer, the links between biological and environmental parameters were weaker than in the upper layers. Thus, the abiotic variables only partially explained the variability of the microbial and viral populations in the deep waters layer (Fig. 5).

## Discussion

### Latitudinal patterns of microbial and viral populations

The microbial abundance in the different depth layers was comparable with previous studies conducted in the North Atlantic Ocean and in the Pacific Ocean (De Corte *et al*., 2010; 2012; Yokokawa *et al*., 2013) exhibiting clear geographic and depth‐related patterns. The latitudinal variability in microbial abundance was not limited to the epipelagic layer but was clearly detectable throughout the whole water column down to the bathypelagic environment (∼6000 m depth).

The tight relationships found between phytoplankton productivity, POC flux and microbial abundance and leucine incorporation throughout the entire water column have also been previously reported for other oceanic regions, such as the North Atlantic Ocean (De Corte *et al*., 2012). These relationships support the notion that the dark ocean's microbial activity mainly depends on sinking particles originating from epipelagic phytoplankton production (Nagata *et al*., 2000; Baltar *et al*., 2009).

The VMR generally increased with depth because of the lower decrease in viral abundance with depth than in microbial abundance. Increasing VMRs with depth were also reported from the open North Atlantic (Parada *et al*., 2007; De Corte *et al*., 2010) and the Pacific (Yang *et al*., 2014).

The oceanic currents and the stratification of the water column (Moore *et al*., 2013) limit the input of nutrients for primary producers into the euphotic layer and conversely, the organic carbon export into the deeper layers in the centre of the gyre. A prevalence of lysogeny and a high burst size have been suggested as viral survival mechanisms under oligotrophic environmental conditions and low host abundance (Weinbauer and Suttle, 1999; Weinbauer *et al*., 2003; Bongiorni *et al*., 2005) similar to those found in the centre of the Southern Atlantic gyre. However, the lack of a significant increase of lysogeny with increasing depth found in the North Atlantic (De Corte *et al*., 2010; 2012) suggests that other viral survival strategies such as pseudo‐lysogeny should be considered as well in the deep ocean. Another possible factor that might contribute to explain the increasing VMR towards the deeper layers of the ocean is the low viral decay rates prevailing in the cold deep waters (Parada *et al*., 2007). Furthermore, a recent study conducted in the Pacific Ocean showed the presence of photosystem II reaction centre genes (*psb*A) of viral origin in the aphotic layers, suggesting a downward flux of viruses from the euphotic zone to the deep ocean on sinking particles (Hurwitz *et al*., 2014; 2015) that could add to the high numbers of viral particles in deep waters as compared with microbial cells.

The three viral populations distinguished by flow cytometry showed a clear depth‐related and geographic pattern (Fig. 3), suggesting (i) that each viral population is likely composed of specific taxonomic groups with a specific genome size, (ii) that each viral population may consist of several taxa able to infect different hosts and (iii) that the clear shift in the viral populations along the transect is probably due to the water masses' circulation in the South Atlantic that shape the host populations and consequently the viral communities.

Recent metagenomic studies on marine viruses conducted in the global surface ocean showed a significant variation of the viral communities between different Longhurst provinces with their characteristic physicochemical conditions and host abundance (Hurwitz *et al*., 2014; Brum *et al*., 2015) in agreement with our results. In contrast, a study on viral morphology reported minimal viral geographic variation (with a dominance of non‐tailed viruses) across six different oceans (Brum *et al*., 2013). This indicates that the viral morphology is considerably less variable than the genomic composition of viral communities in the ocean.

The tendency of the high‐fluorescence viral population to co‐vary with the eukaryotic phytoplankton abundance has been reported for coastal waters (Brussaard, 2004b; Brussaard *et al*., 2008). Only recently, however, sorting the different viral populations (high, medium and low fluorescence) and the subsequent molecular analyses revealed that these viral populations are taxonomically different (Martinez‐Martinez *et al*., 2014). Particularly, the V_HNA and the V_MNA populations mostly contained sequences of cyanophages and eukaryotic algal viruses, thus confirming the findings of Brussaard and colleagues (2004b, 2008). In contrast, the V_LNA populations mostly comprised bacteriophages such as *Myoviridae* and *Podoviridae* families infecting mainly the bacterial clades SAR11 and SAR116 (Martinez‐Martinez *et al*., 2014). This is in accordance with the high abundance of these two morphotypes in the euphotic zones of the ocean (Brum *et al*., 2013).

### Virus–host interactions

Recent studies conducted in surface waters describe a tight relation between microbial and viral abundance and production both in coastal and in open oceans (Winter *et al*., 2004; 2005; Parada *et al*., 2008). This positive correlation has been generally interpreted as an indication of a direct link between viruses and their hosts. Thus, it has been assumed that the viral distribution pattern is mainly determined by microbial abundance. However, studies conducted in the deep ocean reported that viruses and microbes are not always tightly coupled (De Corte *et al*., 2012; Yang *et al*., 2014). The results obtained by path analysis support the notion that the viral abundance in the epipelagic and mesopelagic layers is mainly influenced by host availability and physicochemical variables (Winter *et al*., 2004; 2005; Parada *et al*., 2008; Yang *et al*., 2010; Brum *et al*., 2013; 2015). In contrast, in the bathypelagic layer, the relationship between microbial and viral populations was weak (Fig. 5). Thus, the variation in viral abundance in the deep Atlantic cannot be comprehensively explained by the biotic and abiotic parameters measured, consistent with results obtained from the deep Pacific Ocean and the Southern Ocean (Yang *et al*., 2014). This latter study revealed that viral abundance decreases with time in the Circumpolar Deep and Pacific Deep Waters, suggesting that the decay rates exceed the viral production rates during the deep‐water mass circulation (Yang *et al*., 2014). This finding might seem contradictory to the high VMR found in the deep ocean. However, the high VMR could be (partly) explained by the input of viruses associated to sinking particles originating in the euphotic layers (Hurwitz *et al*., 2015). Several lines of evidence suggest that microbes in the deep ocean are mainly attached to particles (Baltar *et al*., 2009; Herndl and Reinthaler, 2013). Thus, the balance between viral production, decay and viral flux associated to sinking particles may contribute to the weak correlations found between the viral populations and their hosts in the deep ocean.

## Conclusion

Our data revealed that picophytoplankton and microbial abundance are the main controlling factors of the viral populations in the epi‐ and mesopelagic layers of the Atlantic Ocean, supporting the notion that the viral distribution in the surface ocean mainly depends on host availability. However, in the bathypelagic realm, viral abundance is only weakly related to host abundance suggesting that other environmental and/or biological variables may control the viral distribution and virus–host interactions in the deep ocean. Additionally, the viral populations distinguished by flow cytometry showed a clear geographic distribution pattern, suggesting that these populations are composed of distinct taxa.

## Supporting information


**Fig. S1.** Relationship between the calculated POC flux and microbial abundance and leucine incorporation rates.

**Fig. S2.** Percentage of high nucleic acid microbes (% HNA) versus depth.

**Fig. S3.** Picophytoplankton abundance measured in the South Atlantic along the WTRA, SATL and SANT province: (A) *Synechococcus* sp., (B) *Prochlorococcus* sp. and (C) photosynthetic picoeukaryotic abundance.

**Fig. S4**. Depth distribution of three viral populations (V_HNA, V_MNA and V_LNA) in the South Atlantic Ocean.

**Table S1.** Microbial and viral parameters at the different stations sampled in the South Atlantic Ocean. Latitude, longitude, depth and temperature are indicated for each individual sample.

**Table S2.** Physicochemical characteristics of the different depth layers (epipelagic, mesopelagic and bathypelagic) in the three oceanographic regions studied. Average, standard deviation (SD) and number of samples (*n*) are indicated.

**Table S3.** Picophytoplankton, microbial and viral parameters determined in the epipelagic, mesopelagic and bathypelagic layers along the South Atlantic latitudinal transect (oceanic provinces as in Fig. 1). ^3^H‐Leu, leucine incorporation rate; HNA, high nucleic acid content microbes; LNA, low nucleic acid content microbes; MA, microbial abundance, n.d., not detected; SPEC_^3^H‐Leu, specific leucine incorporation rate; VMR, virus‐to‐microbe ratio; V_HNA, high nucleic acid content viruses; V_LNA, low nucleic acid content viruses; V_MNA, medium nucleic acid content viruses.
