# Supplementary figures and images for: Large‐scale distribution of microbial and viral populations in the South Atlantic Ocean
Source: Environ Microbiol Rep. 2016 Feb 16;8(2):305–15. doi: 10.1111/1758-2229.12381 (PMC4959534; doi:10.1111/1758-2229.12381)

Figure.S1

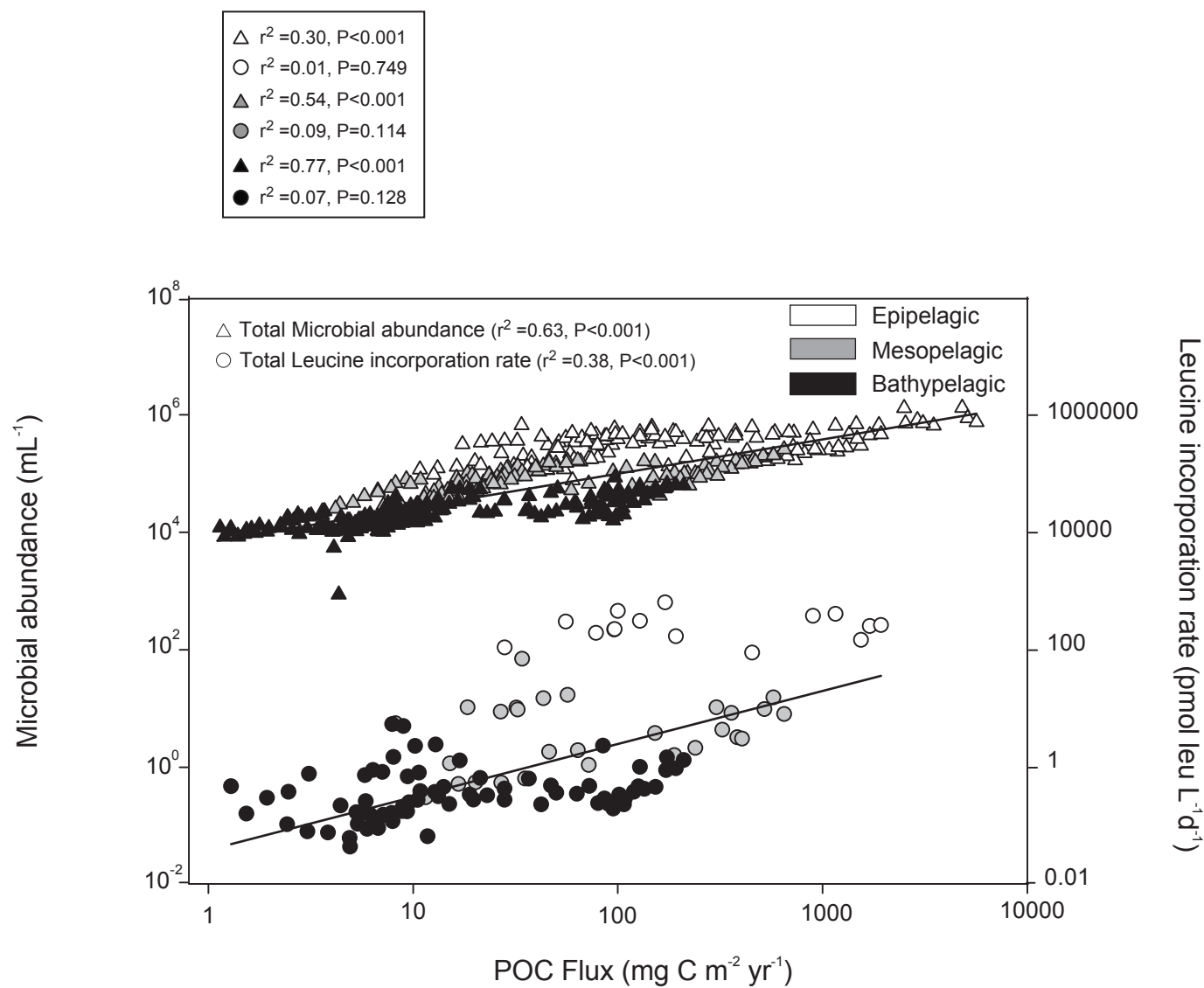

Supplement: Supplementary file 1 — Fig. S1. Relationship between the calculated POC flux and microbial abundance and leucine incorporation rates. [file EMI4-8-305-s001.pdf]

Figure.S2

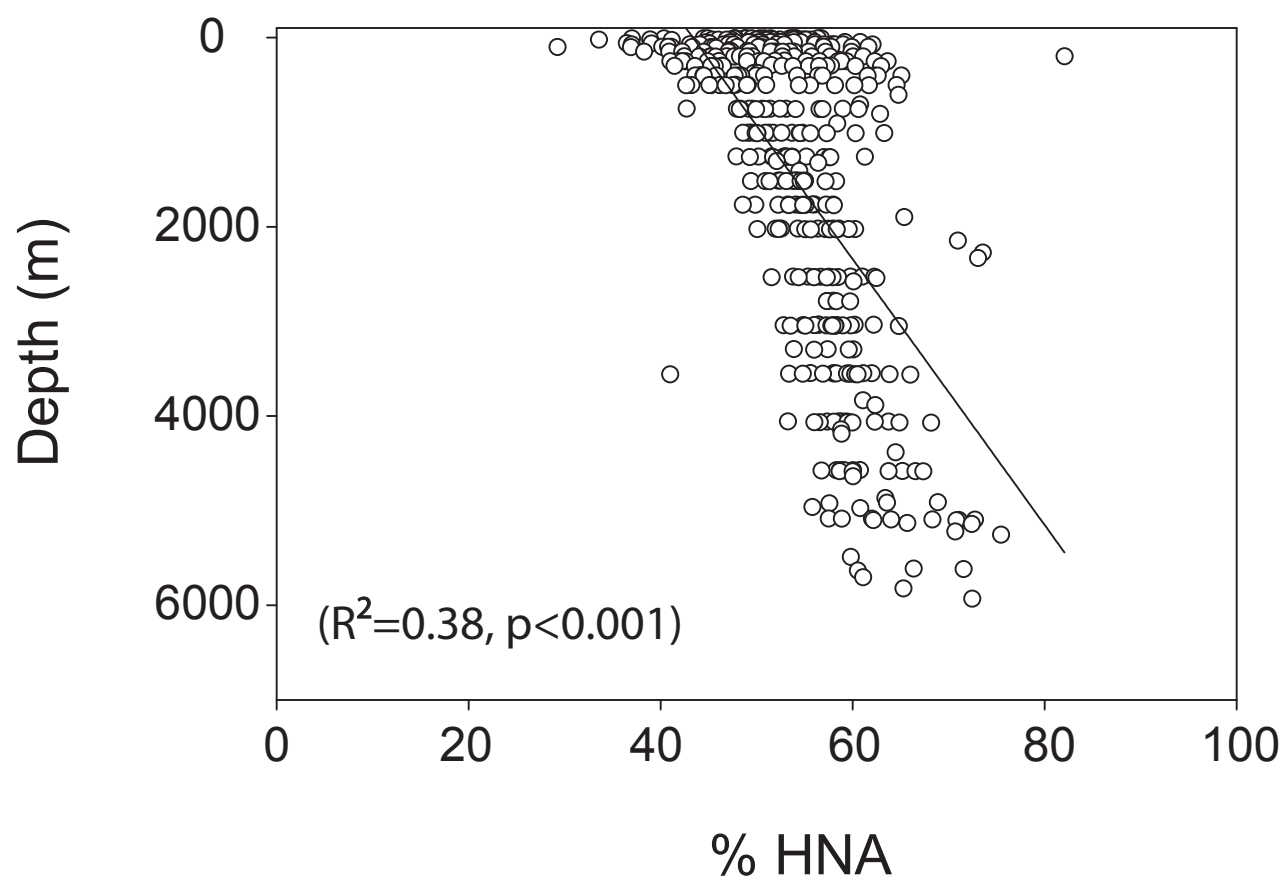

Supplement: Supplementary file 2 — Fig. S2. Percentage of high nucleic acid microbes (% HNA) versus depth. [file EMI4-8-305-s002.pdf]

Figure.S3

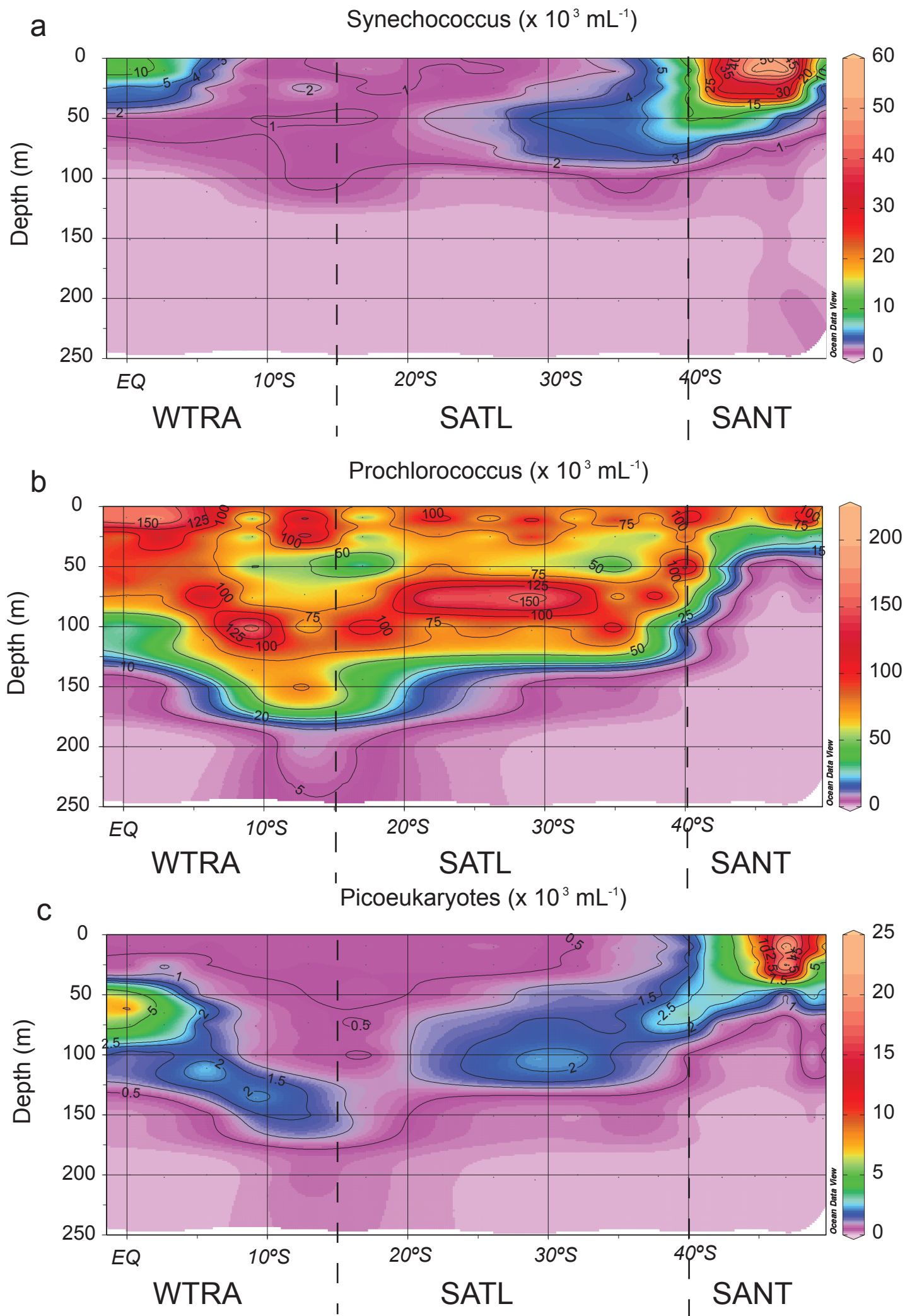

Supplement: Supplementary file 3 — Fig. S3. Picophytoplankton abundance measured in the South Atlantic along the WTRA, SATL and SANT province: (A) Synechococcus sp., (B) Prochlorococcus sp. and (C) photosynthetic picoeukaryotic abundance. [file EMI4-8-305-s003.pdf]

Figure.S4

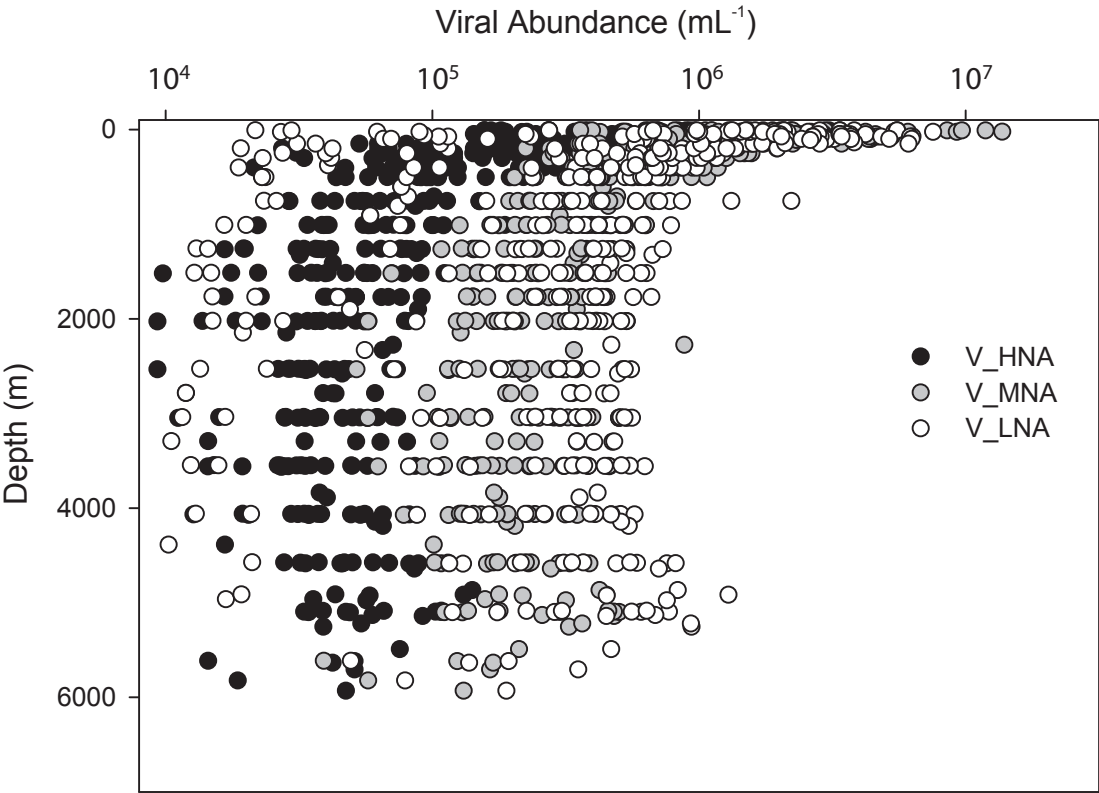

Supplement: Supplementary file 4 — Fig. S4. Depth distribution of three viral populations (V_HNA, V_MNA and V_LNA) in the South Atlantic Ocean. [file EMI4-8-305-s004.pdf]
