# Supplementary material for: Large‐scale distribution of microbial and viral populations in the South Atlantic Ocean
Source: Environ Microbiol Rep. 2016 Feb 16;8(2):305–15. doi: 10.1111/1758-2229.12381 (PMC4959534; doi:10.1111/1758-2229.12381)
Supplement: Supplementary file 5 — Table S1. Microbial and viral parameters at the different stations sampled in the South Atlantic Ocean. Latitude, longitude, depth and temperature are indicated for each individual sample. [file EMI4-8-305-s005.docx]

**Table S1.** Microbial and viral parameters at the different stations sampled in the South Atlantic Ocean. Latitude, longitude, depth and temperature are indicated for each individual sample.

| St | Latitude | Longitude | Depth (m) | Temp (ºC) | PA (x10^5^ mL^-1^) | VA (x105 mL^-1^) | ^3^H-Leu (pmol L^-1^ d^-1^) |
| --- | --- | --- | --- | --- | --- | --- | --- |
| 1 | -49.5475 | -52.6885 | 10 | 10.00 | 6.57 | 1.37 |  |
| 1 | -49.5475 | -52.6885 | 24 | 9.52 | 6.67 | 1.55 |  |
| 1 | -49.5475 | -52.6885 | 50 | 8.30 | 6.56 | 2.48 | 410.65 |
| 1 | -49.5475 | -52.6885 | 76 | 5.58 | 3.60 | 1.95 |  |
| 1 | -49.5475 | -52.6885 | 100 | 4.39 | 2.86 | 1.61 |  |
| 1 | -49.5475 | -52.6885 | 153 | 3.74 | 1.63 | 1.07 |  |
| 1 | -49.5475 | -52.6885 | 202 | 3.38 | 1.49 | 1.04 |  |
| 1 | -49.5475 | -52.6885 | 252 | 3.13 | 1.45 | 0.80 | 3.24 |
| 1 | -49.5475 | -52.6885 | 402 | 2.84 | 1.10 | 0.71 |  |
| 1 | -49.5475 | -52.6885 | 403 | 2.86 | 1.14 | 0.56 |  |
| 1 | -49.5475 | -52.6885 | 504 | 2.81 | 1.02 | 0.62 | 2.12 |
| 1 | -49.5475 | -52.6885 | 606 | 2.72 | 1.00 | 0.59 |  |
| 1 | -49.5475 | -52.6885 | 709 | 2.72 | 1.02 | 0.68 |  |
| 1 | -49.5475 | -52.6885 | 808 | 2.67 | 0.93 | 0.62 |  |
| 1 | -49.5475 | -52.6885 | 909 | 2.60 | 0.42 | 0.42 |  |
| 1 | -49.5475 | -52.6885 | 1010 | 2.48 | 0.86 | 0.63 |  |
| 1 | -49.5475 | -52.6885 | 1263 | 2.29 | 0.62 | 0.53 | 1.01 |
| 1 | -49.5475 | -52.6885 | 1520 | 2.15 | 0.46 | 0.49 |  |
| 1 | -49.5475 | -52.6885 | 1773 | 1.98 | 0.45 | 0.37 | 0.34 |
| 1 | -49.5475 | -52.6885 | 1900 | 1.90 | 0.86 | 0.49 |  |
| 1 | -49.5475 | -52.6885 | 2025 | 1.78 | 0.41 | 0.21 |  |
| 1 | -49.5475 | -52.6885 | 2147 | 1.64 | 0.41 | 0.18 |  |
| 1 | -49.5475 | -52.6885 | 2275 | 1.38 | 0.53 | 1.42 |  |
| 1 | -49.5475 | -52.6885 | 2331 | 1.27 | 0.45 | 0.47 | 2.33 |
| 2 | -48.9358 | -48.7933 | 11 | 11.27 | 7.14 | 4.71 |  |
| 2 | -48.9358 | -48.7933 | 25 | 9.84 | 6.93 | 2.97 |  |
| 2 | -48.9358 | -48.7933 | 51 | 7.84 | 5.61 | 3.69 | 377.93 |
| 2 | -48.9358 | -48.7933 | 76 | 6.12 | 5.07 | 2.79 |  |
| 2 | -48.9358 | -48.7933 | 100 | 5.06 | 4.62 | 1.38 |  |
| 2 | -48.9358 | -48.7933 | 152 | 3.87 | 2.45 | 0.80 |  |
| 2 | -48.9358 | -48.7933 | 204 | 3.50 | 1.96 | 0.63 |  |
| 2 | -48.9358 | -48.7933 | 252 | 3.14 | 1.60 | 0.81 | 10.47 |
| 2 | -48.9358 | -48.7933 | 305 | 2.87 | 1.37 | 0.66 |  |
| 2 | -48.9358 | -48.7933 | 406 | 3.03 | 1.02 | 0.52 |  |
| 2 | -48.9358 | -48.7933 | 506 | 2.97 | 1.02 | 0.48 | 1.60 |
| 2 | -48.9358 | -48.7933 | 758 | 2.88 | 0.70 | 0.54 |  |
| 2 | -48.9358 | -48.7933 | 1013 | 2.72 | 0.62 | 0.48 |  |
| 2 | -48.9358 | -48.7933 | 1266 | 2.64 | 0.54 | 0.39 | 0.29 |
| 2 | -48.9358 | -48.7933 | 1520 | 2.48 | 0.52 | 0.40 |  |
| 2 | -48.9358 | -48.7933 | 2028 | 2.39 | 0.40 | 0.34 |  |
| 2 | -48.9358 | -48.7933 | 2542 | 1.94 | 0.30 | 0.28 | 0.35 |
| 2 | -48.9358 | -48.7933 | 3050 | 1.32 | 0.29 | 0.26 |  |
| 2 | -48.9358 | -48.7933 | 3564 | 0.96 | 0.23 | 0.27 | 0.37 |
| 2 | -48.9358 | -48.7933 | 4074 | 0.58 | 0.21 | 0.29 |  |
| 2 | -48.9358 | -48.7933 | 4588 | 0.38 | 0.18 | 0.37 | 0.23 |
| 2 | -48.9358 | -48.7933 | 5104 | 0.33 | 0.21 | 0.35 |  |
| 2 | -48.9358 | -48.7933 | 5620 | 0.35 | 0.24 | 0.36 | 0.64 |
| 2 | -48.9358 | -48.7933 | 5931 | 0.37 | 0.23 | 0.36 |  |
| 3 | -46.907 | -47.179 | 10 | 17.17 | 13.23 | 14.70 |  |
| 3 | -46.907 | -47.179 | 25 | 16.82 | 13.08 | 10.30 |  |
| 3 | -46.907 | -47.179 | 51 | 14.84 | 2.98 | 3.68 | 147.29 |
| 3 | -46.907 | -47.179 | 75 | 14.12 | 2.37 | 2.34 |  |
| 3 | -46.907 | -47.179 | 102 | 13.72 | 2.51 | 2.13 |  |
| 3 | -46.907 | -47.179 | 152 | 12.61 | 1.69 | 1.95 |  |
| 3 | -46.907 | -47.179 | 202 | 10.60 | 1.78 | 2.28 |  |
| 3 | -46.907 | -47.179 | 252 | 8.75 | 1.90 | 2.28 | 9.69 |
| 3 | -46.907 | -47.179 | 303 | 7.07 | 1.74 | 1.92 |  |
| 3 | -46.907 | -47.179 | 404 | 4.89 | 1.69 | 1.81 |  |
| 3 | -46.907 | -47.179 | 506 | 4.30 | 1.27 | 1.70 | 4.36 |
| 3 | -46.907 | -47.179 | 758 | 3.50 | 0.98 | 1.07 |  |
| 3 | -46.907 | -47.179 | 1012 | 2.99 | 0.82 | 0.32 |  |
| 3 | -46.907 | -47.179 | 1266 | 2.83 | 0.68 | 0.28 | 1.47 |
| 3 | -46.907 | -47.179 | 1521 | 2.73 | 0.55 | 0.20 |  |
| 3 | -46.907 | -47.179 | 2029 | 2.65 | 0.49 | 0.16 | 0.47 |
| 3 | -46.907 | -47.179 | 2535 | 2.56 | 0.37 | 0.13 | 0.29 |
| 3 | -46.907 | -47.179 | 3048 | 2.01 | 0.33 | 0.16 |  |
| 3 | -46.907 | -47.179 | 3560 | 1.37 | 0.24 | 0.16 | 0.29 |
| 3 | -46.907 | -47.179 | 4072 | 0.93 | 0.23 | 0.19 |  |
| 3 | -46.907 | -47.179 | 4586 | 0.50 | 0.18 | 0.26 | 0.48 |
| 3 | -46.907 | -47.179 | 5103 | 0.35 | 0.17 | 0.26 |  |
| 3 | -46.907 | -47.179 | 5616 | 0.33 | 0.26 | 0.10 |  |
| 3 | -46.907 | -47.179 | 5824 | 0.33 | 0.32 | 0.16 |  |
| 4 | -44.7117 | -45.5265 | 10 | 16.97 | 8.82 | 20.50 |  |
| 4 | -44.7117 | -45.5265 | 23 | 16.30 | 8.12 | 22.50 |  |
| 4 | -44.7117 | -45.5265 | 51 | 11.90 | 4.51 | 4.81 | 253.32 |
| 4 | -44.7117 | -45.5265 | 75 | 9.09 | 2.81 | 5.06 |  |
| 4 | -44.7117 | -45.5265 | 100 | 8.63 | 2.54 | 5.23 |  |
| 4 | -44.7117 | -45.5265 | 150 | 8.13 | 2.24 | 3.41 |  |
| 4 | -44.7117 | -45.5265 | 201 | 6.64 | 1.99 | 2.47 |  |
| 4 | -44.7117 | -45.5265 | 252 | 5.35 | 2.08 | 2.46 | 15.36 |
| 4 | -44.7117 | -45.5265 | 299 | 4.80 | 2.02 | 1.86 |  |
| 4 | -44.7117 | -45.5265 | 405 | 4.10 | 1.53 | 1.64 |  |
| 4 | -44.7117 | -45.5265 | 505 | 3.62 | 1.21 | 1.30 | 8.42 |
| 4 | -44.7117 | -45.5265 | 758 | 3.01 | 0.96 | 0.58 |  |
| 4 | -44.7117 | -45.5265 | 1012 | 2.83 | 0.63 | 1.00 |  |
| 4 | -44.7117 | -45.5265 | 1264 | 2.73 | 0.64 | 0.83 | 0.96 |
| 4 | -44.7117 | -45.5265 | 1519 | 2.72 | 0.56 | 0.71 |  |
| 4 | -44.7117 | -45.5265 | 1772 | 2.73 | 0.58 | 0.71 | 0.46 |
| 4 | -44.7117 | -45.5265 | 2028 | 2.64 | 0.45 | 0.62 |  |
| 4 | -44.7117 | -45.5265 | 2537 | 2.24 | 0.32 | 0.50 | 0.38 |
| 4 | -44.7117 | -45.5265 | 3047 | 1.54 | 0.26 | 0.57 |  |
| 4 | -44.7117 | -45.5265 | 3560 | 0.97 | 0.16 | 0.22 | 0.20 |
| 4 | -44.7117 | -45.5265 | 4068 | 0.52 | 0.20 | 0.54 |  |
| 4 | -44.7117 | -45.5265 | 4584 | 0.31 | 0.20 | 0.68 | 0.24 |
| 4 | -44.7117 | -45.5265 | 5101 | 0.26 | 0.35 | 0.90 |  |
| 4 | -44.7117 | -45.5265 | 5142 | 0.27 | 0.30 | 1.02 |  |
| 5 | -42.364 | -44.0318 | 10 | 17.21 | 7.56 | 17.30 |  |
| 5 | -42.364 | -44.0318 | 25 | 16.32 | 7.22 | 18.90 |  |
| 5 | -42.364 | -44.0318 | 51 | 13.46 | 4.69 | 9.01 | 264.15 |
| 5 | -42.364 | -44.0318 | 75 | 10.72 | 3.91 | 5.75 |  |
| 5 | -42.364 | -44.0318 | 102 | 9.64 | 2.83 | 3.75 |  |
| 5 | -42.364 | -44.0318 | 152 | 9.08 | 2.61 | 2.55 |  |
| 5 | -42.364 | -44.0318 | 202 | 8.18 | 2.48 | 2.20 |  |
| 5 | -42.364 | -44.0318 | 252 | 6.48 | 2.58 | 2.05 | 8.03 |
| 5 | -42.364 | -44.0318 | 300 | 5.40 | 2.05 | 2.20 |  |
| 5 | -42.364 | -44.0318 | 404 | 4.50 | 1.59 | 1.93 |  |
| 5 | -42.364 | -44.0318 | 505 | 4.00 | 1.46 | 1.51 | 3.05 |
| 5 | -42.364 | -44.0318 | 758 | 3.23 | 0.92 | 1.41 |  |
| 5 | -42.364 | -44.0318 | 1011 | 2.93 | 0.85 | 1.18 |  |
| 5 | -42.364 | -44.0318 | 1325 | 2.70 | 0.64 | 1.06 | 1.32 |
| 5 | -42.364 | -44.0318 | 1518 | 2.74 | 0.63 | 1.04 |  |
| 5 | -42.364 | -44.0318 | 1772 | 2.63 | 0.58 | 1.03 | 0.89 |
| 5 | -42.364 | -44.0318 | 2025 | 2.60 | 0.47 | 0.91 |  |
| 5 | -42.364 | -44.0318 | 2535 | 2.40 | 0.35 | 0.86 | 0.43 |
| 5 | -42.364 | -44.0318 | 3048 | 1.63 | 0.32 | 0.84 |  |
| 5 | -42.364 | -44.0318 | 3558 | 1.05 | 0.20 | 0.72 | 0.23 |
| 5 | -42.364 | -44.0318 | 4072 | 0.55 | 0.19 | 0.89 |  |
| 5 | -42.364 | -44.0318 | 4584 | 0.32 | 0.19 | 1.13 | 0.24 |
| 5 | -42.364 | -44.0318 | 5099 | 0.26 | 0.26 | 0.95 |  |
| 5 | -42.364 | -44.0318 | 5257 | 0.27 | 0.29 | 1.30 |  |
| 6 | -39.9648 | -42.4232 | 10 | 21.26 | 4.72 | 7.67 |  |
| 6 | -39.9648 | -42.4232 | 25 | 21.32 | 4.95 | 7.40 |  |
| 6 | -39.9648 | -42.4232 | 51 | 18.96 | 6.18 | 8.60 | 89.37 |
| 6 | -39.9648 | -42.4232 | 66 | 16.82 | 4.46 | 7.15 |  |
| 6 | -39.9648 | -42.4232 | 102 | 13.05 | 2.27 | 2.80 |  |
| 6 | -39.9648 | -42.4232 | 150 | 9.99 | 1.88 | 3.37 |  |
| 6 | -39.9648 | -42.4232 | 202 | 8.79 | 1.33 | 1.77 |  |
| 6 | -39.9648 | -42.4232 | 252 | 7.35 | 1.58 | 2.37 | 3.82 |
| 6 | -39.9648 | -42.4232 | 302 | 6.27 | 1.62 | 2.28 |  |
| 6 | -39.9648 | -42.4232 | 404 | 5.20 | 1.25 | 1.78 |  |
| 6 | -39.9648 | -42.4232 | 505 | 4.42 | 1.09 | 1.31 |  |
| 6 | -39.9648 | -42.4232 | 756 | 3.69 | 0.68 | 1.16 | 1.09 |
| 6 | -39.9648 | -42.4232 | 1010 | 3.20 | 0.52 | 0.98 |  |
| 6 | -39.9648 | -42.4232 | 1264 | 2.91 | 0.56 | 0.93 |  |
| 6 | -39.9648 | -42.4232 | 1412 | 2.89 | 0.47 | 0.85 | 0.49 |
| 6 | -39.9648 | -42.4232 | 2027 | 3.08 | 0.41 | 0.76 |  |
| 6 | -39.9648 | -42.4232 | 3045 | 1.67 | 0.36 | 0.78 |  |
| 6 | -39.9648 | -42.4232 | 3046 | 1.67 | 0.35 | 0.93 | 0.43 |
| 6 | -39.9648 | -42.4232 | 3047 | 1.67 | 0.35 | 0.92 | 0.28 |
| 6 | -39.9648 | -42.4232 | 3558 | 1.06 | 0.23 | 0.92 |  |
| 6 | -39.9648 | -42.4232 | 4064 | 0.55 | 0.21 | 0.78 | 0.33 |
| 6 | -39.9648 | -42.4232 | 4582 | 0.30 | 0.22 | 1.15 |  |
| 6 | -39.9648 | -42.4232 | 5097 | 0.26 | 0.40 | 1.10 | 0.28 |
| 6 | -39.9648 | -42.4232 | 5221 | 0.26 | 0.42 | 1.35 |  |
| 7 | -37.8382 | -41.1305 | 10 | 20.42 | 5.05 | 6.46 |  |
| 7 | -37.8382 | -41.1305 | 24 | 20.40 | 4.35 | 5.71 |  |
| 7 | -37.8382 | -41.1305 | 50 | 19.54 | 5.08 | 7.57 | 169.19 |
| 7 | -37.8382 | -41.1305 | 74 | 16.54 | 5.58 | 12.90 |  |
| 7 | -37.8382 | -41.1305 | 101 | 15.71 | 3.28 | 5.40 |  |
| 7 | -37.8382 | -41.1305 | 151 | 15.22 | 2.29 | 2.75 |  |
| 7 | -37.8382 | -41.1305 | 201 | 14.72 | 1.77 | 1.95 |  |
| 7 | -37.8382 | -41.1305 | 252 | 14.27 | 1.67 | 1.67 | 1.93 |
| 7 | -37.8382 | -41.1305 | 303 | 13.73 | 1.53 | 1.28 |  |
| 7 | -37.8382 | -41.1305 | 404 | 10.91 | 1.55 | 1.51 | 1.83 |
| 7 | -37.8382 | -41.1305 | 504 | 8.85 | 1.28 | 1.70 |  |
| 7 | -37.8382 | -41.1305 | 757 | 4.62 | 0.79 | 1.22 |  |
| 7 | -37.8382 | -41.1305 | 1012 | 3.67 | 0.68 | 1.33 |  |
| 7 | -37.8382 | -41.1305 | 1265 | 3.11 | 0.54 | 1.28 | 0.65 |
| 7 | -37.8382 | -41.1305 | 1518 | 2.89 | 0.53 | 0.99 |  |
| 7 | -37.8382 | -41.1305 | 1770 | 2.88 | 0.56 | 1.14 | 1.29 |
| 7 | -37.8382 | -41.1305 | 2024 | 2.90 | 0.55 | 0.94 |  |
| 7 | -37.8382 | -41.1305 | 2534 | 2.60 | 0.37 | 0.82 | 0.32 |
| 7 | -37.8382 | -41.1305 | 3042 | 2.05 | 0.31 | 0.76 |  |
| 7 | -37.8382 | -41.1305 | 3555 | 1.24 | 0.30 | 0.82 | 0.28 |
| 7 | -37.8382 | -41.1305 | 4068 | 0.65 | 0.21 | 0.63 |  |
| 7 | -37.8382 | -41.1305 | 4585 | 0.31 | 0.17 | 0.77 | 0.18 |
| 7 | -37.8382 | -41.1305 | 5097 | 0.25 | 0.41 | 1.14 |  |
| 7 | -37.8382 | -41.1305 | 5132 | 0.26 | 0.34 | 0.99 |  |
| 8 | -35.0083 | -39.4425 | 11 | 22.09 | 4.06 | 5.21 |  |
| 8 | -35.0083 | -39.4425 | 25 | 22.04 | 4.06 | 5.05 |  |
| 8 | -35.0083 | -39.4425 | 50 | 21.95 | 4.22 | 5.87 | 639.53 |
| 8 | -35.0083 | -39.4425 | 75 | 17.66 | 4.31 | 9.30 |  |
| 8 | -35.0083 | -39.4425 | 101 | 16.82 | 4.71 | 9.00 |  |
| 8 | -35.0083 | -39.4425 | 152 | 16.33 | 1.86 | 3.48 |  |
| 8 | -35.0083 | -39.4425 | 202 | 15.84 | 1.62 | 3.32 |  |
| 8 | -35.0083 | -39.4425 | 250 | 15.37 | 1.64 | 3.00 | 16.99 |
| 8 | -35.0083 | -39.4425 | 301 | 14.74 | 1.29 | 1.79 |  |
| 8 | -35.0083 | -39.4425 | 403 | 13.34 | 1.11 | 1.73 |  |
| 8 | -35.0083 | -39.4425 | 504 | 11.16 | 0.90 | 1.37 | 0.64 |
| 8 | -35.0083 | -39.4425 | 756 | 5.17 | 0.66 | 1.89 |  |
| 8 | -35.0083 | -39.4425 | 1009 | 3.88 | 0.56 | 1.17 |  |
| 8 | -35.0083 | -39.4425 | 1264 | 3.19 | 0.36 | 0.91 | 0.34 |
| 8 | -35.0083 | -39.4425 | 1518 | 2.92 | 0.34 | 1.11 |  |
| 8 | -35.0083 | -39.4425 | 1770 | 3.00 | 0.31 | 0.83 | 0.24 |
| 8 | -35.0083 | -39.4425 | 2025 | 3.08 | 0.24 | 0.81 |  |
| 8 | -35.0083 | -39.4425 | 2532 | 3.14 | 0.19 | 0.77 | 0.07 |
| 8 | -35.0083 | -39.4425 | 3042 | 2.79 | 0.15 | 0.69 |  |
| 8 | -35.0083 | -39.4425 | 3555 | 1.75 | 0.15 | 0.56 | 0.18 |
| 8 | -35.0083 | -39.4425 | 4067 | 0.71 | 0.17 | 0.91 |  |
| 8 | -35.0083 | -39.4425 | 4579 | 0.30 | 0.18 | 0.86 | 0.17 |
| 8 | -35.0083 | -39.4425 | 4869 | 0.24 | 0.31 | 1.41 |  |
| 8 | -35.0083 | -39.4425 | 4918 | 0.25 | 0.30 | 1.87 |  |
| 9 | -32.092 | -37.4563 | 10 | 23.84 | 4.03 | 3.87 |  |
| 9 | -32.092 | -37.4563 | 26 | 23.72 | 4.40 | 3.60 |  |
| 9 | -32.092 | -37.4563 | 51 | 20.45 | 5.83 | 6.29 | 226.04 |
| 9 | -32.092 | -37.4563 | 75 | 18.51 | 5.28 | 10.50 |  |
| 9 | -32.092 | -37.4563 | 102 | 17.28 | 4.92 | 6.55 |  |
| 9 | -32.092 | -37.4563 | 151 | 16.16 | 1.68 | 2.49 |  |
| 9 | -32.092 | -37.4563 | 200 | 15.44 | 1.50 | 2.23 |  |
| 9 | -32.092 | -37.4563 | 252 | 14.95 | 1.35 | 2.12 | 9.57 |
| 9 | -32.092 | -37.4563 | 303 | 14.40 | 1.20 | 2.01 |  |
| 9 | -32.092 | -37.4563 | 405 | 12.90 | 0.95 | 1.25 |  |
| 9 | -32.092 | -37.4563 | 505 | 10.82 | 0.83 | 1.19 | 0.55 |
| 9 | -32.092 | -37.4563 | 759 | 5.68 | 0.51 | 0.94 |  |
| 9 | -32.092 | -37.4563 | 1011 | 3.96 | 0.39 | 0.86 |  |
| 9 | -32.092 | -37.4563 | 1261 | 3.22 | 0.27 | 0.70 | 0.39 |
| 9 | -32.092 | -37.4563 | 1517 | 2.95 | 0.23 | 0.71 | 0.25 |
| 9 | -32.092 | -37.4563 | 1770 | 2.90 | 0.20 | 0.64 |  |
| 9 | -32.092 | -37.4563 | 2022 | 3.03 | 0.19 | 0.46 |  |
| 9 | -32.092 | -37.4563 | 2534 | 3.03 | 0.18 | 0.74 | 0.09 |
| 9 | -32.092 | -37.4563 | 2786 | 2.89 | 0.13 | 0.56 |  |
| 9 | -32.092 | -37.4563 | 3043 | 2.71 | 0.13 | 0.72 | 0.09 |
| 9 | -32.092 | -37.4563 | 3300 | 2.26 | 0.16 | 0.71 |  |
| 9 | -32.092 | -37.4563 | 3554 | 1.86 | 0.13 | 0.78 | 0.11 |
| 9 | -32.092 | -37.4563 | 4144 | 0.58 | 0.16 | 0.76 |  |
| 9 | -32.092 | -37.4563 | 4190 | 0.49 | 0.16 | 0.81 |  |
| 10 | -29.0538 | -35.8078 | 11 | 24.10 | 4.07 |  |  |
| 10 | -29.0538 | -35.8078 | 25 | 24.00 | 3.90 | 4.34 |  |
| 10 | -29.0538 | -35.8078 | 51 | 20.13 | 5.05 | 7.81 | 310.39 |
| 10 | -29.0538 | -35.8078 | 76 | 18.22 | 4.66 | 7.74 |  |
| 10 | -29.0538 | -35.8078 | 103 | 16.95 | 3.07 | 6.26 |  |
| 10 | -29.0538 | -35.8078 | 151 | 15.95 | 1.76 | 3.20 |  |
| 10 | -29.0538 | -35.8078 | 204 | 15.23 | 1.48 | 2.10 |  |
| 10 | -29.0538 | -35.8078 | 251 | 14.60 | 1.37 | 2.13 | 14.88 |
| 10 | -29.0538 | -35.8078 | 302 | 14.01 | 1.09 | 1.76 |  |
| 10 | -29.0538 | -35.8078 | 404 | 11.98 | 0.90 | 2.17 |  |
| 10 | -29.0538 | -35.8078 | 504 | 9.82 | 0.80 | 1.32 | 0.55 |
| 10 | -29.0538 | -35.8078 | 757 | 5.49 | 0.50 | 0.80 |  |
| 10 | -29.0538 | -35.8078 | 1011 | 3.65 | 0.38 | 0.91 |  |
| 10 | -29.0538 | -35.8078 | 1306 | 3.05 | 0.25 | 0.97 | 0.46 |
| 10 | -29.0538 | -35.8078 | 1515 | 2.92 | 0.18 | 0.69 | 0.38 |
| 10 | -29.0538 | -35.8078 | 1770 | 2.85 | 0.16 | 0.74 |  |
| 10 | -29.0538 | -35.8078 | 2025 | 2.89 | 0.15 | 0.61 |  |
| 10 | -29.0538 | -35.8078 | 2579 | 2.89 | 0.15 | 0.76 | 0.21 |
| 10 | -29.0538 | -35.8078 | 2789 | 2.80 | 0.13 | 0.72 |  |
| 10 | -29.0538 | -35.8078 | 3044 | 2.67 | 0.14 | 0.67 | 0.12 |
| 10 | -29.0538 | -35.8078 | 3299 | 2.43 | 0.12 | 0.89 |  |
| 10 | -29.0538 | -35.8078 | 3555 | 2.05 | 0.10 | 0.66 | 0.16 |
| 10 | -29.0538 | -35.8078 | 3836 | 1.45 | 0.10 | 0.63 |  |
| 10 | -29.0538 | -35.8078 | 3888 | 1.41 | 0.11 | 0.57 |  |
| 11 | -26.0902 | -34.2812 | 10 | 25.91 | 3.50 | 3.50 |  |
| 11 | -26.0902 | -34.2812 | 25 | 25.91 | 3.48 | 3.29 |  |
| 11 | -26.0902 | -34.2812 | 52 | 22.88 | 3.20 | 7.37 | 191.99 |
| 11 | -26.0902 | -34.2812 | 74 | 21.82 | 3.43 | 6.50 |  |
| 11 | -26.0902 | -34.2812 | 101 | 21.35 | 2.75 | 4.52 |  |
| 11 | -26.0902 | -34.2812 | 151 | 19.97 | 1.89 | 2.63 |  |
| 11 | -26.0902 | -34.2812 | 202 | 17.35 | 1.39 | 2.33 |  |
| 11 | -26.0902 | -34.2812 | 252 | 16.03 | 1.12 | 2.50 | 8.81 |
| 11 | -26.0902 | -34.2812 | 303 | 15.05 | 0.96 | 1.80 |  |
| 11 | -26.0902 | -34.2812 | 403 | 12.89 | 0.95 | 1.76 |  |
| 11 | -26.0902 | -34.2812 | 506 | 10.63 | 0.74 | 1.23 | 0.52 |
| 11 | -26.0902 | -34.2812 | 756 | 5.73 | 0.50 | 1.39 |  |
| 11 | -26.0902 | -34.2812 | 1006 | 3.82 | 0.30 | 0.89 |  |
| 11 | -26.0902 | -34.2812 | 1258 | 3.14 | 0.21 | 0.95 | 4.99 |
| 11 | -26.0902 | -34.2812 | 1515 | 3.28 | 0.19 | 0.75 | 5.42 |
| 11 | -26.0902 | -34.2812 | 1771 | 3.37 | 0.15 | 0.85 |  |
| 11 | -26.0902 | -34.2812 | 2024 | 3.34 | 0.16 | 0.82 |  |
| 11 | -26.0902 | -34.2812 | 2533 | 3.01 | 0.16 | 0.81 | 0.17 |
| 11 | -26.0902 | -34.2812 | 2787 | 2.87 | 0.14 | 0.71 |  |
| 11 | -26.0902 | -34.2812 | 3043 | 2.73 | 0.14 | 0.92 | 0.04 |
| 11 | -26.0902 | -34.2812 | 3297 | 2.59 | 0.12 | 0.74 |  |
| 11 | -26.0902 | -34.2812 | 3553 | 2.32 | 0.11 | 0.83 | 0.22 |
| 11 | -26.0902 | -34.2812 | 4067 | 1.35 | 0.10 | 0.71 |  |
| 11 | -26.0902 | -34.2812 | 4640 | 0.29 | 0.23 | 1.07 |  |
| 12 | -22.4732 | -32.7487 | 10 | 26.38 | 3.13 | 2.94 |  |
| 12 | -22.4732 | -32.7487 | 23 | 26.34 | 3.29 | 2.71 |  |
| 12 | -22.4732 | -32.7487 | 50 | 24.71 | 4.21 | 6.76 | 220.47 |
| 12 | -22.4732 | -32.7487 | 76 | 22.41 | 1.76 | 11.90 |  |
| 12 | -22.4732 | -32.7487 | 99 | 21.67 | 0.77 | 9.57 |  |
| 12 | -22.4732 | -32.7487 | 151 | 19.51 | 1.15 | 4.90 |  |
| 12 | -22.4732 | -32.7487 | 200 | 17.64 | 1.09 | 3.86 |  |
| 12 | -22.4732 | -32.7487 | 251 | 16.15 | 1.23 | 2.07 | 10.29 |
| 12 | -22.4732 | -32.7487 | 300 | 14.50 | 0.86 | 1.58 |  |
| 12 | -22.4732 | -32.7487 | 401 | 12.55 | 0.66 | 1.25 |  |
| 12 | -22.4732 | -32.7487 | 504 | 10.02 | 0.64 | 1.38 |  |
| 12 | -22.4732 | -32.7487 | 755 | 5.22 | 0.35 | 0.78 | 1.16 |
| 12 | -22.4732 | -32.7487 | 1009 | 3.66 | 0.28 | 0.84 |  |
| 12 | -22.4732 | -32.7487 | 1263 | 3.44 | 0.21 | 0.69 | 0.81 |
| 12 | -22.4732 | -32.7487 | 1516 | 3.88 | 0.17 | 0.62 | 0.69 |
| 12 | -22.4732 | -32.7487 | 1769 | 3.71 | 0.18 | 0.72 |  |
| 12 | -22.4732 | -32.7487 | 2021 | 3.42 | 0.17 | 0.88 |  |
| 12 | -22.4732 | -32.7487 | 2531 | 3.01 | 0.13 | 0.55 | 0.11 |
| 12 | -22.4732 | -32.7487 | 2785 | 2.87 | 0.13 | 0.76 |  |
| 12 | -22.4732 | -32.7487 | 3040 | 2.75 | 0.13 | 0.69 | 0.26 |
| 12 | -22.4732 | -32.7487 | 3295 | 2.61 | 0.12 | 0.55 |  |
| 12 | -22.4732 | -32.7487 | 3551 | 2.31 | 0.11 | 0.54 | 0.17 |
| 12 | -22.4732 | -32.7487 | 4064 | 1.35 | 0.08 | 0.64 |  |
| 12 | -22.4732 | -32.7487 | 4575 | 0.45 | 0.18 | 0.84 |  |
| 13 | -17.0173 | -30.6052 | 9 | 27.90 | 3.49 | 2.72 |  |
| 13 | -17.0173 | -30.6052 | 25 | 27.88 | 3.72 | 2.51 |  |
| 13 | -17.0173 | -30.6052 | 49 | 27.64 | 4.04 | 3.93 | 303.81 |
| 13 | -17.0173 | -30.6052 | 74 | 25.04 | 4.26 | 5.55 |  |
| 13 | -17.0173 | -30.6052 | 100 | 23.72 | 2.03 | 12.30 |  |
| 13 | -17.0173 | -30.6052 | 144 | 22.14 | 2.23 | 3.76 |  |
| 13 | -17.0173 | -30.6052 | 202 | 18.30 | 1.41 | 2.44 |  |
| 13 | -17.0173 | -30.6052 | 251 | 15.88 | 1.02 | 1.95 | 10.44 |
| 13 | -17.0173 | -30.6052 | 301 | 13.76 | 0.85 | 1.73 |  |
| 13 | -17.0173 | -30.6052 | 403 | 10.48 | 0.59 | 1.27 |  |
| 13 | -17.0173 | -30.6052 | 502 | 8.03 | 0.45 | 1.13 | 0.31 |
| 13 | -17.0173 | -30.6052 | 755 | 4.49 | 0.29 | 2.55 |  |
| 13 | -17.0173 | -30.6052 | 1009 | 3.77 | 0.29 | 0.68 |  |
| 13 | -17.0173 | -30.6052 | 1260 | 3.95 | 0.20 | 0.62 | 0.16 |
| 13 | -17.0173 | -30.6052 | 1514 | 3.93 | 0.17 | 0.59 |  |
| 13 | -17.0173 | -30.6052 | 1766 | 3.48 | 0.16 | 0.57 | 0.06 |
| 13 | -17.0173 | -30.6052 | 2022 | 3.25 | 0.14 | 0.57 |  |
| 13 | -17.0173 | -30.6052 | 2529 | 2.87 | 0.11 | 0.58 | 0.08 |
| 13 | -17.0173 | -30.6052 | 3040 | 2.67 | 0.11 | 0.56 |  |
| 13 | -17.0173 | -30.6052 | 3551 | 2.44 | 0.12 | 0.52 | 0.08 |
| 13 | -17.0173 | -30.6052 | 4061 | 1.93 | 0.09 | 0.49 |  |
| 13 | -17.0173 | -30.6052 | 4575 | 1.09 | 0.11 | 0.58 |  |
| 13 | -17.0173 | -30.6052 | 4926 | 0.44 | 0.18 | 0.73 |  |
| 13 | -17.0173 | -30.6052 | 4976 | 0.43 | 0.16 | 1.13 | 0.11 |
| 14 | -12.8957 | -29.2335 | 10 | 28.11 | 2.93 | 1.81 |  |
| 14 | -12.8957 | -29.2335 | 24 | 27.75 | 3.04 | 1.88 |  |
| 14 | -12.8957 | -29.2335 | 50 | 27.27 | 3.66 | 2.22 | 109.08 |
| 14 | -12.8957 | -29.2335 | 73 | 25.60 | 3.38 | 4.74 |  |
| 14 | -12.8957 | -29.2335 | 100 | 24.47 | 3.17 | 5.06 |  |
| 14 | -12.8957 | -29.2335 | 150 | 22.82 | 1.24 | 10.70 |  |
| 14 | -12.8957 | -29.2335 | 201 | 17.02 | 1.18 | 2.35 |  |
| 14 | -12.8957 | -29.2335 | 252 | 13.67 | 0.80 | 1.45 |  |
| 14 | -12.8957 | -29.2335 | 302 | 11.99 | 0.69 | 1.18 | 5.57 |
| 14 | -12.8957 | -29.2335 | 403 | 8.74 | 0.52 | 0.88 |  |
| 14 | -12.8957 | -29.2335 | 504 | 6.71 | 0.41 | 0.72 |  |
| 14 | -12.8957 | -29.2335 | 756 | 4.62 | 0.30 | 0.62 |  |
| 14 | -12.8957 | -29.2335 | 1009 | 3.97 | 0.23 | 0.58 |  |
| 14 | -12.8957 | -29.2335 | 1261 | 4.09 | 0.19 | 0.47 | 0.77 |
| 14 | -12.8957 | -29.2335 | 1513 | 4.17 | 0.20 | 0.51 |  |
| 14 | -12.8957 | -29.2335 | 1767 | 3.76 | 0.17 | 0.49 | 0.38 |
| 14 | -12.8957 | -29.2335 | 2021 | 3.32 | 0.13 | 0.57 |  |
| 14 | -12.8957 | -29.2335 | 2530 | 2.83 | 0.10 | 0.44 | 0.30 |
| 14 | -12.8957 | -29.2335 | 3037 | 2.64 | 0.10 | 0.40 |  |
| 14 | -12.8957 | -29.2335 | 3549 | 2.41 | 0.11 | 0.54 | 0.16 |
| 14 | -12.8957 | -29.2335 | 4062 | 1.77 | 0.08 | 0.43 |  |
| 14 | -12.8957 | -29.2335 | 4576 | 1.05 | 0.10 | 0.46 | 0.47 |
| 14 | -12.8957 | -29.2335 | 5088 | 0.76 | 0.08 | 0.47 |  |
| 14 | -12.8957 | -29.2335 | 5492 | 0.61 | 0.12 | 0.75 |  |
| 15 | -9.1608 | -28.0012 | 10 | 28.78 | 3.10 | 1.20 |  |
| 15 | -9.1608 | -28.0012 | 25 | 28.65 | 3.40 | 1.35 |  |
| 15 | -9.1608 | -28.0012 | 50 | 28.09 | 3.64 | 1.10 |  |
| 15 | -9.1608 | -28.0012 | 76 | 25.76 | 3.52 | 5.05 |  |
| 15 | -9.1608 | -28.0012 | 101 | 24.32 | 1.62 | 5.90 |  |
| 15 | -9.1608 | -28.0012 | 136 | 21.68 | 1.31 | 6.37 |  |
| 15 | -9.1608 | -28.0012 | 199 | 13.82 | 0.96 | 1.62 |  |
| 15 | -9.1608 | -28.0012 | 248 | 11.36 | 0.79 | 1.28 |  |
| 15 | -9.1608 | -28.0012 | 292 | 9.82 | 0.67 | 1.05 |  |
| 15 | -9.1608 | -28.0012 | 402 | 7.86 | 0.57 | 1.01 |  |
| 15 | -9.1608 | -28.0012 | 502 | 6.79 | 0.48 | 0.75 |  |
| 15 | -9.1608 | -28.0012 | 755 | 4.82 | 0.32 | 0.64 |  |
| 15 | -9.1608 | -28.0012 | 1008 | 4.06 | 0.25 | 0.55 |  |
| 15 | -9.1608 | -28.0012 | 1260 | 4.23 | 0.20 | 0.46 |  |
| 15 | -9.1608 | -28.0012 | 1515 | 4.12 | 0.19 | 0.53 |  |
| 15 | -9.1608 | -28.0012 | 1768 | 3.81 | 0.18 | 0.50 |  |
| 15 | -9.1608 | -28.0012 | 2021 | 3.41 | 0.16 | 0.43 |  |
| 15 | -9.1608 | -28.0012 | 2529 | 2.97 | 0.12 | 0.45 |  |
| 15 | -9.1608 | -28.0012 | 3038 | 2.73 | 0.12 | 0.47 |  |
| 15 | -9.1608 | -28.0012 | 3549 | 2.52 | 0.13 | 0.70 |  |
| 15 | -9.1608 | -28.0012 | 4059 | 1.83 | 0.11 | 0.55 |  |
| 15 | -9.1608 | -28.0012 | 4574 | 1.03 | 0.09 | 0.46 |  |
| 15 | -9.1608 | -28.0012 | 5088 | 0.80 | 0.09 | 0.49 |  |
| 15 | -9.1608 | -28.0012 | 5707 | 0.68 | 0.12 | 0.57 |  |
| 16 | -5.6768 | -28.4595 | 10 | 28.88 | 4.36 | 3.50 |  |
| 16 | -5.6768 | -28.4595 | 25 | 28.85 | 3.98 | 3.70 |  |
| 16 | -5.6768 | -28.4595 | 50 | 28.66 | 4.31 | 3.57 |  |
| 16 | -5.6768 | -28.4595 | 74 | 27.80 | 4.07 | 9.26 |  |
| 16 | -5.6768 | -28.4595 | 111 | 21.00 | 2.74 | 5.81 |  |
| 16 | -5.6768 | -28.4595 | 150 | 16.23 | 1.09 | 2.42 |  |
| 16 | -5.6768 | -28.4595 | 200 | 11.36 | 6.69 | 1.43 |  |
| 16 | -5.6768 | -28.4595 | 251 | 10.21 | 1.28 | 1.70 |  |
| 16 | -5.6768 | -28.4595 | 301 | 9.41 | 0.88 | 1.34 |  |
| 16 | -5.6768 | -28.4595 | 378 | 8.42 | 0.70 | 1.23 |  |
| 16 | -5.6768 | -28.4595 | 504 | 6.59 | 0.70 | 0.88 |  |
| 16 | -5.6768 | -28.4595 | 756 | 5.00 | 0.45 | 0.74 |  |
| 16 | -5.6768 | -28.4595 | 1008 | 4.17 | 0.39 | 0.75 |  |
| 16 | -5.6768 | -28.4595 | 1260 | 4.35 | 0.29 | 0.53 |  |
| 16 | -5.6768 | -28.4595 | 1514 | 4.21 | 0.24 | 0.56 |  |
| 16 | -5.6768 | -28.4595 | 1767 | 3.92 | 0.23 | 0.54 |  |
| 16 | -5.6768 | -28.4595 | 2020 | 3.42 | 0.21 | 0.47 |  |
| 16 | -5.6768 | -28.4595 | 2530 | 2.88 | 0.16 | 0.43 |  |
| 16 | -5.6768 | -28.4595 | 3036 | 2.68 | 0.15 | 0.59 |  |
| 16 | -5.6768 | -28.4595 | 3550 | 2.52 | 0.14 | 0.50 |  |
| 16 | -5.6768 | -28.4595 | 4061 | 1.83 | 0.11 | 0.51 |  |
| 16 | -5.6768 | -28.4595 | 4574 | 0.95 | 0.11 | 0.50 |  |
| 16 | -5.6768 | -28.4595 | 5087 | 0.80 | 0.13 | 1.20 |  |
| 16 | -5.6768 | -28.4595 | 5636 | 0.72 | 0.13 | 0.35 |  |
| 17 | -2.6505 | -28.9172 | 10 | 28.78 | 6.41 | 0.59 |  |
| 17 | -2.6505 | -28.9172 | 26 | 28.63 | 6.27 | 0.68 |  |
| 17 | -2.6505 | -28.9172 | 65 | 21.08 | 4.60 |  |  |
| 17 | -2.6505 | -28.9172 | 75 | 19.57 | 3.45 | 1.91 |  |
| 17 | -2.6505 | -28.9172 | 100 | 14.86 | 2.22 | 1.09 |  |
| 17 | -2.6505 | -28.9172 | 150 | 13.46 | 1.26 | 0.44 |  |
| 17 | -2.6505 | -28.9172 | 202 | 12.81 | 1.29 | 0.53 |  |
| 17 | -2.6505 | -28.9172 | 251 | 12.17 | 1.13 | 0.57 |  |
| 17 | -2.6505 | -28.9172 | 301 | 11.70 | 0.98 | 0.60 |  |
| 17 | -2.6505 | -28.9172 | 377 | 10.38 | 0.91 | 0.58 |  |
| 17 | -2.6505 | -28.9172 | 503 | 7.98 | 0.66 | 0.39 |  |
| 17 | -2.6505 | -28.9172 | 754 | 4.93 | 0.53 | 0.29 |  |
| 17 | -2.6505 | -28.9172 | 1008 | 4.38 | 0.38 | 0.28 |  |
| 17 | -2.6505 | -28.9172 | 1259 | 4.49 | 0.30 | 0.16 |  |
| 17 | -2.6505 | -28.9172 | 1514 | 4.19 | 0.23 | 0.16 |  |
| 17 | -2.6505 | -28.9172 | 1768 | 3.85 | 0.22 | 0.27 |  |
| 17 | -2.6505 | -28.9172 | 2020 | 3.67 | 0.20 | 0.25 |  |
| 17 | -2.6505 | -28.9172 | 2529 | 2.93 | 0.17 | 0.19 |  |
| 17 | -2.6505 | -28.9172 | 3038 | 2.63 | 0.13 | 0.21 |  |
| 17 | -2.6505 | -28.9172 | 3549 | 2.47 | 0.12 | 0.18 |  |
| 17 | -2.6505 | -28.9172 | 4061 | 1.93 | 0.12 | 0.23 |  |
| 17 | -2.6505 | -28.9172 | 4572 | 0.97 | 0.01 | 0.23 |  |
| 17 | -2.6505 | -28.9172 | 4914 | 0.78 | 0.10 | 0.24 |  |
| 17 | -2.6505 | -28.9172 | 4964 | 0.77 | 0.05 | 0.21 |  |
| 18 | -0.1915 | -32.8745 | 9 | 28.61 | 5.15 | 0.54 |  |
| 18 | -0.1915 | -32.8745 | 25 | 28.44 | 5.28 | 1.79 |  |
| 18 | -0.1915 | -32.8745 | 62 | 25.82 | 3.85 | 1.55 | 459.16 |
| 18 | -0.1915 | -32.8745 | 75 | 25.71 | 4.36 | 1.76 |  |
| 18 | -0.1915 | -32.8745 | 100 | 17.60 | 1.80 | 1.00 |  |
| 18 | -0.1915 | -32.8745 | 151 | 14.03 | 1.19 | 0.52 |  |
| 18 | -0.1915 | -32.8745 | 200 | 13.06 | 1.17 | 0.27 |  |
| 18 | -0.1915 | -32.8745 | 246 | 12.16 | 1.13 | 0.34 |  |
| 18 | -0.1915 | -32.8745 | 302 | 10.71 | 1.07 | 0.33 | 69.74 |
| 18 | -0.1915 | -32.8745 | 403 | 8.09 | 0.94 | 0.27 |  |
| 18 | -0.1915 | -32.8745 | 503 | 7.00 | 0.76 | 0.29 |  |
| 18 | -0.1915 | -32.8745 | 756 | 5.25 | 0.56 | 0.29 |  |
| 18 | -0.1915 | -32.8745 | 1008 | 4.49 | 0.38 | 0.29 |  |
| 18 | -0.1915 | -32.8745 | 1261 | 4.59 | 0.28 | 0.18 | 2.43 |
| 18 | -0.1915 | -32.8745 | 1514 | 4.27 | 0.24 | 0.19 |  |
| 18 | -0.1915 | -32.8745 | 1766 | 3.94 | 0.26 | 0.17 | 2.30 |
| 18 | -0.1915 | -32.8745 | 2020 | 3.52 | 0.23 | 0.17 |  |
| 18 | -0.1915 | -32.8745 | 2529 | 3.02 | 0.21 | 0.27 | 1.49 |
| 18 | -0.1915 | -32.8745 | 2784 | 2.81 | 0.19 | 0.12 |  |
| 18 | -0.1915 | -32.8745 | 3039 | 2.67 | 0.15 | 0.14 | 0.83 |
| 18 | -0.1915 | -32.8745 | 3292 | 2.55 | 0.18 | 0.13 |  |
| 18 | -0.1915 | -32.8745 | 3547 | 2.44 | 0.16 | 0.14 | 0.90 |
| 18 | -0.1915 | -32.8745 | 4059 | 2.15 | 0.19 | 0.16 | 0.73 |
| 18 | -0.1915 | -32.8745 | 4387 | 1.20 | 0.14 | 0.13 |  |
|  |  |  |  |  |  |  |  |
