# Supplementary material for: Large‐scale distribution of microbial and viral populations in the South Atlantic Ocean
Source: Environ Microbiol Rep. 2016 Feb 16;8(2):305–15. doi: 10.1111/1758-2229.12381 (PMC4959534; doi:10.1111/1758-2229.12381)
Supplement: Supplementary file 6 — Table S2. Physicochemical characteristics of the different depth layers (epipelagic, mesopelagic and bathypelagic) in the three oceanographic regions studied. Average, standard deviation (SD) and number of samples (n) are indicated. [file EMI4-8-305-s006.docx]

|  |  |  | WTRA | | | SATL | | | SANT | | |
| --- | --- | --- | --- | --- | --- | --- | --- | --- | --- | --- | --- |
|  |  |  |  |  |  |  |  |  |  |  |  |
| Layers | Depth | Variables | Average | SD | n | Average | SD | n | Average | SD | n |
|  |  |  |  |  |  |  |  |  |  |  |  |
| Epipelagic | 10-200 m | Temperature ºC | 22.83 | 5.93 | 35 | 20.03 | 4.27 | 55 | 10.12 | 4.27 | 34 |
|  |  | Salinity | 36.21 | 0.63 | 35 | 35.98 | 0.64 | 55 | 34.44 | 0.43 | 34 |
|  |  | AOU (μmol kg^-1^) | 39.33 | 58.47 | 35 | 5.92 | 17.53 | 55 | 14.37 | 18.44 | 34 |
| Mesopelagic | 200-1000 m | Temperature ºC | 7.91 | 2.92 | 30 | 9.47 | 4.47 | 47 | 3.76 | 1.43 | 32 |
|  |  | Salinity | 34.75 | 0.25 | 30 | 34.85 | 0.51 | 47 | 34.26 | 0.14 | 32 |
|  |  | AOU (μmol kg^-1^) | 165.15 | 19.32 | 30 | 70.03 | 33.56 | 47 | 86.47 | 39.59 | 32 |
| Bathypelagic | 1000-6000 m | Temperature ºC | 2.64 | 1.22 | 56 | 2.21 | 1.10 | 87 | 1.51 | 0.94 | 51 |
|  |  | Salinity | 34.86 | 0.09 | 56 | 34.76 | 0.13 | 87 | 34.69 | 0.06 | 51 |
|  |  | AOU (μmol kg^-1^) | 97.70 | 20.43 | 56 | 113.3 | 23.05 | 87 | 139.22 | 10.95 | 51 |

Table S2. Physico-chemical characteristics of the different depth layers (epipelagic, mesopelagic and bathypelagic) in the three oceanographic regions studied. Average, standard deviation (SD) and number of samples (n) are indicated.
