# Supplementary material for: Large‐scale distribution of microbial and viral populations in the South Atlantic Ocean
Source: Environ Microbiol Rep. 2016 Feb 16;8(2):305–15. doi: 10.1111/1758-2229.12381 (PMC4959534; doi:10.1111/1758-2229.12381)
Supplement: Supplementary file 7 — Table S3. Picophytoplankton, microbial and viral parameters determined in the epipelagic, mesopelagic and bathypelagic layers along the South Atlantic latitudinal transect (oceanic provinces as in Fig. 1). 3H‐Leu, leucine incorporation rate; HNA, high nucleic acid content microbes; LNA, low nucleic acid content microbes; MA, microbial abundance, n.d., not detected; SPEC_3H‐Leu, specific leucine incorporation rate; VMR, virus‐to‐microbe ratio; V_HNA, high nucleic acid content viruses; V_LNA, low nucleic acid content viruses; V_MNA, medium nucleic acid content viruses. [file EMI4-8-305-s007.docx]

|  |  |  | WTRA | | | SATL | | | SANT | | |
| --- | --- | --- | --- | --- | --- | --- | --- | --- | --- | --- | --- |
|  |  |  |  |  |  |  |  |  |  |  |  |
| Layer | Depth range | Variable | Average | SD | n | Average | SD | n | Average | SD | n |
|  |  |  |  |  |  |  |  |  |  |  |  |
| **Epipelagic** | **10-200 m** | MA (x10^5^ mL^-1^) | 3.19 | 1.60 | 35 | 3.31 | 1.41 | 55 | 4.66 | 2.98 | 34 |
|  |  | HNA (x10^5^ mL^-1^) | 1.67 | 1.03 | 35 | 1.60 | 0.71 | 55 | 2.26 | 1.17 | 34 |
|  |  | LNA (x10^5^ mL^-1^) | 1.51 | 0.76 | 35 | 1.71 | 0.75 | 55 | 2.40 | 1.88 | 34 |
|  |  | %HNA | 51.47 | 8.11 | 35 | 47.90 | 5.46 | 55 | 51.58 | 6.89 | 34 |
|  |  | %LNA | 48.62 | 8.11 | 35 | 52.09 | 5.46 | 55 | 48.41 | 6.89 | 34 |
|  |  | Viruses (x10^5^ mL^-1^) | 27.56 | 24.97 | 35 | 53.76 | 27.97 | 55 | 53.95 | 59.19 | 34 |
|  |  | V_HNA (x10^5^ mL^-1^) | 4.08 | 3.34 | 34 | 7.87 | 5.03 | 54 | 7.34 | 8.23 | 34 |
|  |  | V_MNA (x10^5^ mL^-1^) | 11.86 | 9.15 | 34 | 21.25 | 11.63 | 54 | 30.48 | 33.88 | 34 |
|  |  | V_LNA (x10^5^ mL^-1^) | 11.59 | 14.04 | 34 | 24.58 | 13.34 | 54 | 16.07 | 18.42 | 34 |
|  |  | %V_HNA | 16.38 | 6.65 | 34 | 13.96 | 4.60 | 54 | 13.88 | 4.32 | 34 |
|  |  | %V_MNA | 51.67 | 17.79 | 34 | 39.25 | 5.50 | 54 | 57.02 | 11.84 | 34 |
|  |  | %V_LNA | 31.82 | 21.89 | 34 | 46.74 | 7.23 | 54 | 29.00 | 13.79 | 34 |
|  |  | VMR | 12.37 | 16.57 | 34 | 19.24 | 17.96 | 54 | 10.91 | 6.92 | 34 |
|  |  | Picoeukaryotes (x10^3^ mL^-1^) | 1.18 | 1.88 | 34 | 0.81 | 0.81 | 54 | 3.30 | 6.02 | 32 |
|  |  | *Prochlorococcus* (x10^3^ mL^-1^) | 69.33 | 59.82 | 34 | 58.09 | 56.73 | 54 | 17.27 | 35.53 | 32 |
|  |  | *Synechococcus* (x10^3^ mL^-1^) | 1.55 | 2.81 | 34 | 1.83 | 2.36 | 54 | 9.47 | 16.69 | 32 |
|  |  | ^3^H-Leu (pmol L^-1^ d^-1^) | 284.12 | 175.03 | 2 | 268.84 | 155.15 | 8 | 290.66 | 94.51 | 5 |
|  |  | SPEC_^3^H-Leu (x10^-5^ fmol cell^-1^ d^-1^) | 7.44 | 4.46 | 2 | 6.08 | 3.85 | 8 | 5.83 | 0.61 | 5 |
| **Mesopelagic** | **200-1000 m** | MA (x10^5^ mL^-1^) | 0.66 | 0.28 | 30 | 0.89 | 0.40 | 47 | 1.26 | 0.49 | 32 |
|  |  | HNA (x10^5^ mL^-1^) | 0.34 | 0.16 | 30 | 0.42 | 0.19 | 47 | 0.74 | 0.28 | 32 |
|  |  | LNA (x10^5^ mL^-1^) | 0.32 | 0.13 | 30 | 0.47 | 0.21 | 47 | 0.51 | 0.21 | 32 |
|  |  | %HNA | 50.72 | 3.77 | 30 | 48.00 | 4.43 | 47 | 59.31 | 3.29 | 32 |
|  |  | %LNA | 49.27 | 3.77 | 30 | 51.99 | 4.43 | 47 | 40.68 | 3.39 | 32 |
|  |  | Viruses (x10^5^ mL^-1^) | 7.28 | 3.90 | 30 | 15.36 | 5,24 | 47 | 11.31 | 6.38 | 32 |
|  |  | V_HNA (x10^5^ mL^-1^) | 0.71 | 0.39 | 30 | 1.53 | 1.07 | 47 | 1.27 | 0.99 | 32 |
|  |  | V_MNA (x10^5^ mL^-1^) | 3.45 | 1.30 | 30 | 6.21 | 2.73 | 47 | 6.48 | 3.84 | 32 |
|  |  | V_LNA (x10^5^ mL^-1^) | 3.12 | 2.70 | 30 | 7.60 | 3.30 | 47 | 3.56 | 2.69 | 32 |
|  |  | %V_HNA | 10.16 | 2.89 | 30 | 9.56 | 4.34 | 47 | 11.15 | 4.00 | 32 |
|  |  | %V_MNA | 55.43 | 21.35 | 30 | 40.33 | 8.15 | 47 | 57.42 | 13.00 | 32 |
|  |  | %V_LNA | 34.30 | 23.15 | 30 | 50.10 | 11.91 | 47 | 31.54 | 16.15 | 32 |
|  |  | VMR | 12.61 | 6.60 | 30 | 19.80 | 10.97 | 47 | 8.83 | 3.27 | 32 |
|  |  | Picoeukaryotes (x10^3^ mL^-1^) | n.d | n.d | n.d | n.d | n.d | n.d | n.d | n.d | n.d |
|  |  | *Prochlorococcus* (x10^3^ mL^-1^) | n.d | n.d | n.d | n.d | n.d | n.d | n.d | n.d | n.d |
|  |  | *Synechococcus* (x10^3^ mL^-1^) | n.d | n.d | n.d | n.d | n.d | n.d | n.d | n.d | n.d |
|  |  | ^3^H-Leu (pmol L^-1^ d^-1^) | 37.65 | 32.08 | 2 | 5.21 | 5.50 | 16 | 6.63 | 4.24 | 10 |
|  |  | SPEC_^3^H-Leu (x10^-5^ fmol cell^-1^ d^-1^) | 36.68 | 28.60 | 2 | 4.25 | 3.94 | 16 | 4.05 | 2.13 | 10 |
| **Bathypelagic** | **1000-6000 m** | MA (x10^5^ mL^-1^) | 0.16 | 0.06 | 56 | 0.22 | 0.11 | 87 | 0.37 | 0.16 | 51 |
|  |  | HNA (x10^5^ mL^-1^) | 0.08 | 0.02 | 56 | 0.12 | 0.60 | 87 | 0.23 | 0.10 | 51 |
|  |  | LNA (x10^5^ mL^-1^) | 0.07 | 0.03 | 56 | 0.09 | 0.06 | 87 | 0.14 | 0.07 | 51 |
|  |  | %HNA | 57.24 | 3.85 | 56 | 57.09 | 4.17 | 87 | 61.71 | 6.97 | 51 |
|  |  | %LNA | 42.75 | 3.85 | 56 | 42.91 | 4.17 | 87 | 38.28 | 6.97 | 51 |
|  |  | Viruses (x10^5^ mL^-1^) | 3.89 | 0.20 | 56 | 8.01 | 2.23 | 87 | 5.36 | 3.37 | 51 |
|  |  | V_HNA (x10^5^ mL^-1^) | 0.38 | 0.17 | 56 | 0.54 | 0.23 | 87 | 0.45 | 0.23 | 51 |
|  |  | V_MNA (x10^5^ mL^-1^) | 1.66 | 0.58 | 56 | 2.64 | 0.87 | 87 | 2.43 | 1.63 | 51 |
|  |  | V_LNA (x10^5^ mL^-1^) | 1.84 | 1.56 | 56 | 4.83 | 1.54 | 87 | 2.47 | 2.13 | 51 |
|  |  | %V_HNA | 10.61 | 3.28 | 56 | 6.70 | 2.05 | 87 | 9.92 | 4.41 | 51 |
|  |  | %V_MNA | 52.05 | 22.44 | 56 | 32.94 | 6.64 | 87 | 45.86 | 13.04 | 51 |
|  |  | %V_LNA | 37.29 | 24.99 | 56 | 60.42 | 7.94 | 87 | 44.19 | 16.01 | 51 |
|  |  | VMR | 33.10 | 36.79 | 56 | 42.39 | 14.42 | 87 | 16.45 | 12.29 | 51 |
|  |  | Picoeukaryotes (x10^3^ mL^-1^) | n.d | n.d | n.d | n.d | n.d | n.d | n.d | n.d | n.d |
|  |  | *Prochlorococcus* (x10^3^ mL^-1^) | n.d | n.d | n.d | n.d | n.d | n.d | n.d | n.d | n.d |
|  |  | *Synechococcus* (x10^3^ mL^-1^) | n.d | n.d | n.d | n.d | n.d | n.d | n.d | n.d | n.d |
|  |  | ^3^H-Leu (pmol L^-1^ d^-1^) | 0.98 | 0.73 | 11 | 0.53 | 1.10 | 40 | 0.60 | 0.50 | 23 |
|  |  | Spec_^3^H-Leu (x10^-5^ fmol cell^-1^ d^-1^) | 5.00 | 2.34 | 11 | 2.46 | 5.44 | 40 | 1.55 | 0.94 | 23 |

**Table S3**. Picophytoplankton, microbial and viral parameters determined in the epipelagic, mesopelagic, and bathypelagic layers along the South Atlantic latitudinal transect (oceanic provinces as in Figure 1). MA-microbial abundance, HNA-high nucleic acid content microbes, LNA-low nucleic acid content microbes, V_HNA-high nucleic acid content viruses, V_MNA-medium nucleic acid content viruses, V_LNA- low nucleic acid content viruses, VMR-viral to microbial ratio, ^3^H-Leu-leucine incorporation rate, SPEC_^3^H-Leu-specific leucine incorporation rate, n.d-not detected.
